# Supplementary material for: Jointly efficient encoding and decoding in neural populations
Source: PLoS Comput Biol. 2024 Jul 10;20(7):e1012240. doi: 10.1371/journal.pcbi.1012240 (PMC11262701; doi:10.1371/journal.pcbi.1012240)
Supplement: S1 Fig — Same as Fig 3, in the case of lognormal distribution over stimuli, π(x)=LN(1,1). Top row: high-distortion, low-rate solution. Bottom row: low-distortion, high-rate solution. (A) Bell-shaped tuning curves of the encoder (probability of neuron i to emit a spike, as a function of x). (B) Comparison between the stimulus distribution, π(x) (green curve), and the generative distribution, pψ(x) = ∑r pψ(x|r)pψ(r) (purple curve). (C) Numerical values of the ELBO, and the distortion and rate terms. (PDF) [file pcbi.1012240.s001.pdf]

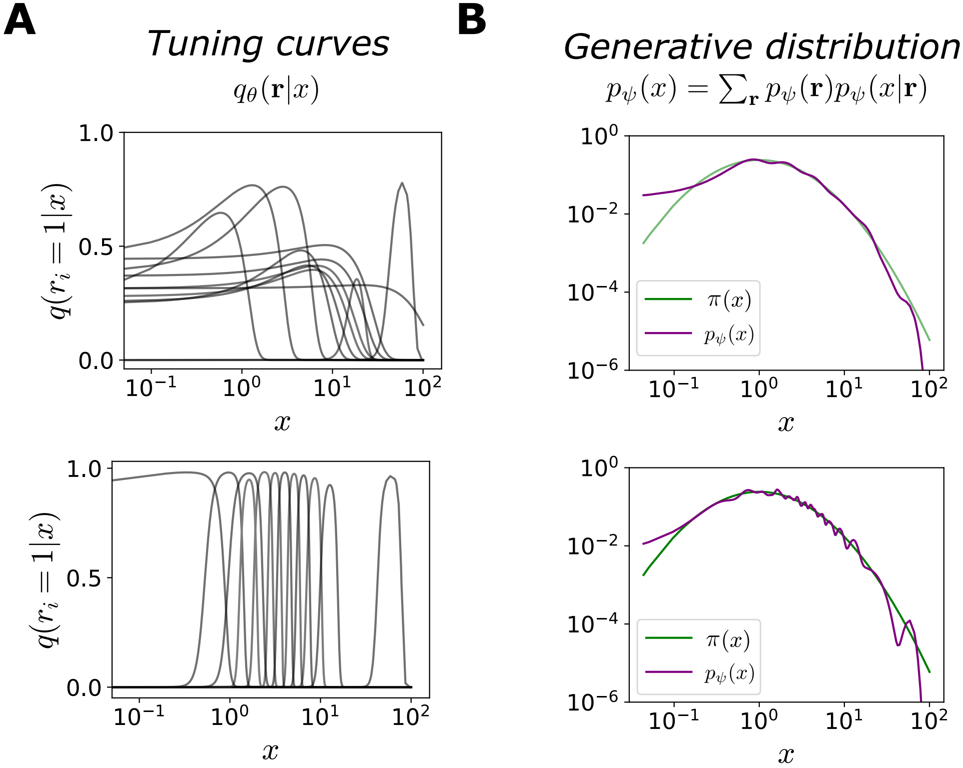

C

Loss functions

ELBO	-2.41
Distortion	1.68
Rate	0.73

ELBO	-2.41
Distortion	0.15
Rate	2.36
