# Supplementary figures and images for: Jointly efficient encoding and decoding in neural populations
Source: PLoS Comput Biol. 2024 Jul 10;20(7):e1012240. doi: 10.1371/journal.pcbi.1012240 (PMC11262701; doi:10.1371/journal.pcbi.1012240)

**A**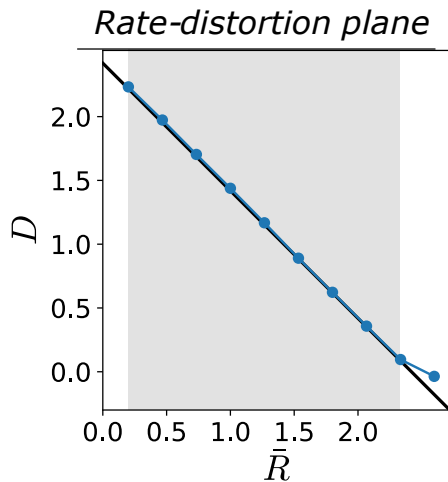**C**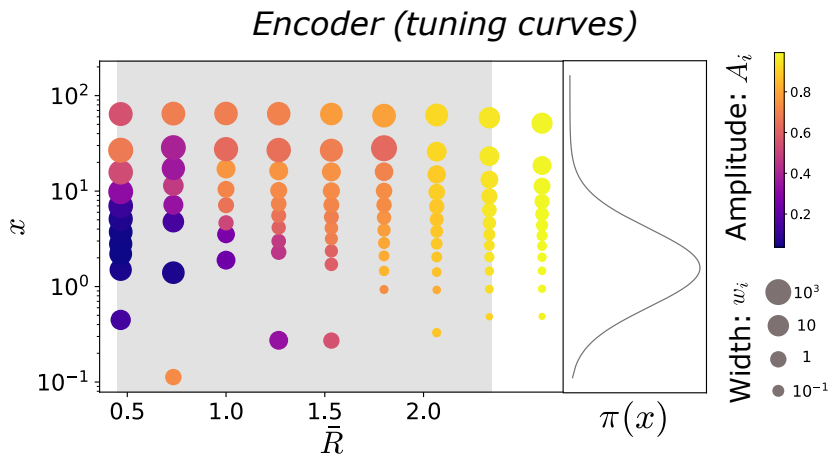**B**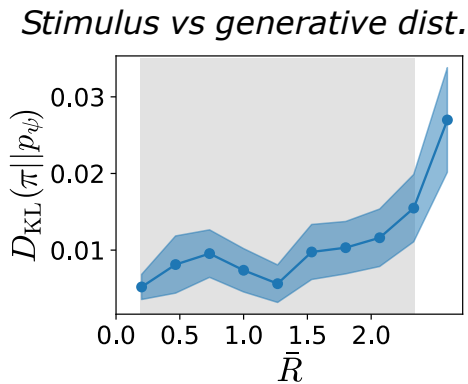**D**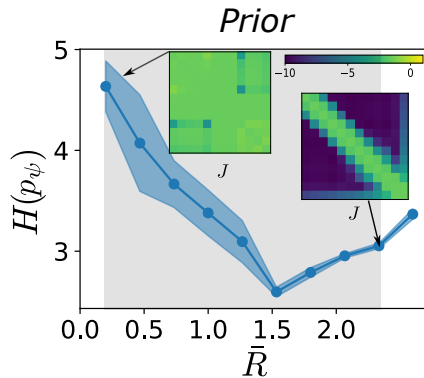**E**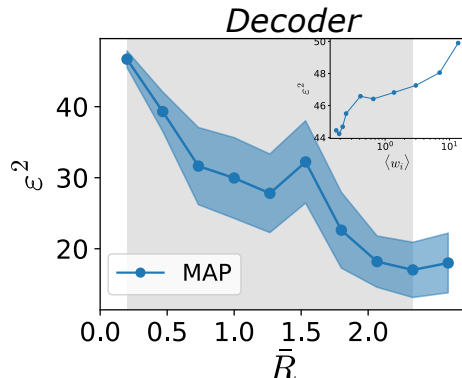

Supplement: S2 Fig — Same as Fig 4, but with a log-normal decoder. (A) Solutions of the ELBO optimization problem as a function of target rate, D(R¯) (blue curve), and theoretical optimum, D = H(π) − R (black curve), in the rate-distortion plane. Values of R¯ where the solutions coincide with the theoretical optimum (grey region). Since the decoder belong to the same parameteric family of the stimulus distribution, it is possible to achieve optimal distortion at very low rates. (B) DKL divergence between the stimulus and the generative distributions, as a function of R¯. (C) Optimal tuning curves for different values of R¯. Each dot represents a neuron: the position on the y-axis corresponds to its preferred stimulus, the size of the dot is proportional to the tuning width, and the color refers to the amplitude (see legend). The curve on the right illustrates the data distribution, π(x). (D) Entropy of the prior distribution over neural activity, pψ(r), as a function of R¯. Insets show two configurations of the coupling matrices, with rows ordered according to the neurons’ preferred stimuli, and coupling strengths colored according to the legend. (E) MSE of the stimulus estimate, obtained as the MAP (blue curve, scale on the left y-axis), or from samples (orange curve, scale on the right y-axis), as a function of R¯. Inset: MSE (MAP) as a function of the average tuning width. (PDF) [file pcbi.1012240.s002.pdf]

**A***Rate-distortion plane*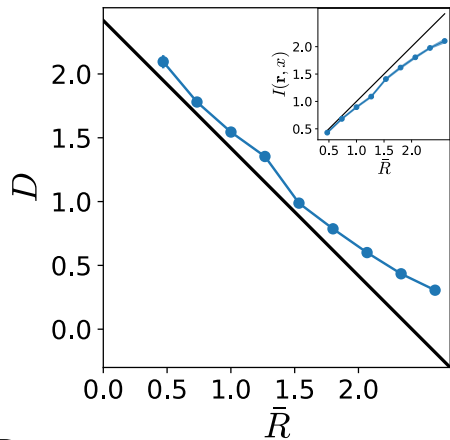**B***Stimulus vs generative dist*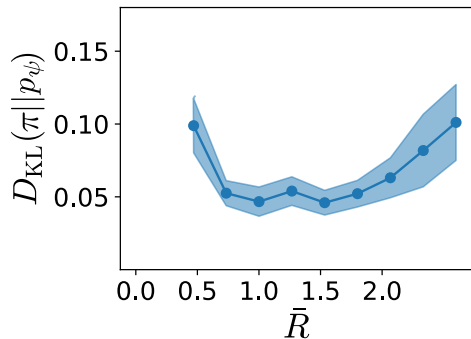**C***Encoder (tuning curves)*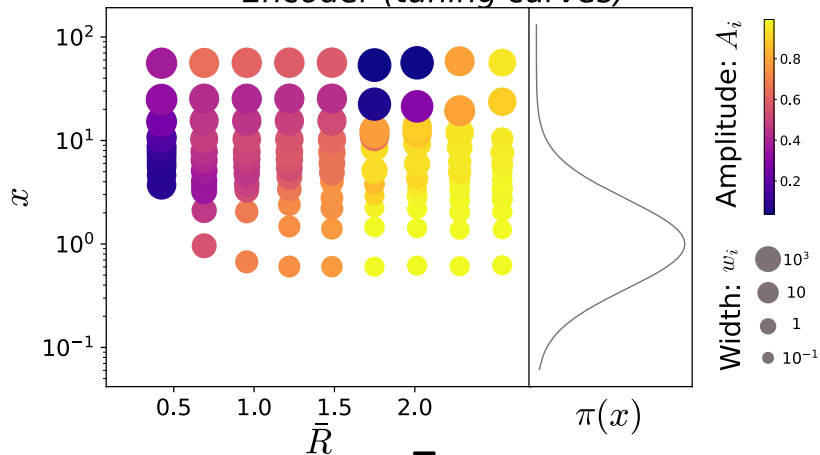**D***Prior*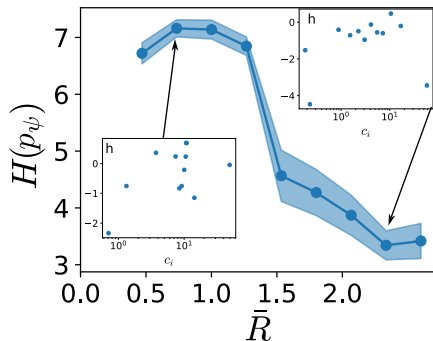**E***Decoder*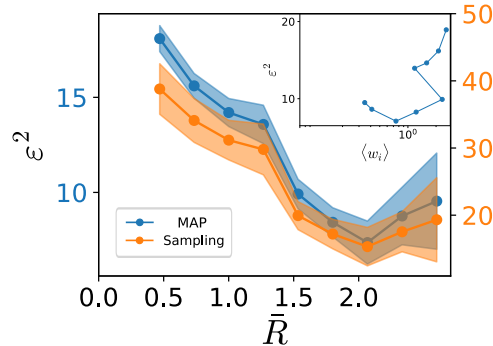

Supplement: S3 Fig — Same as Fig 4, but with pψ(r) a product of independent Bernoulli distributions. The decoder is Gaussian. (A) Solutions of the ELBO optimization problem as a function of target rate, D(R¯) (blue curve), and theoretical optimum, D = H(π) − R (black curve), in the rate-distortion plane. Values of R¯ where the solutions coincide with the theoretical optimum (grey region). Solutions always depart from the optimal line, especially at very high rate, due to the limited flexibility of the prior over neural activity. Inset: mutual information between stimuli and neural responses as a function of R¯. (B) DKL divergence between the stimulus and the generative distributions, as a function of R¯. (C) Optimal tuning curves for different values of R¯. Each dot represents a neuron: the position on the y-axis corresponds to its preferred stimulus, the size of the dot is proportional to the tuning width, and the color refers to the amplitude (see legend). The curve on the right illustrates the data distribution, π(x). (D) Entropy of the prior distribution over neural activity, pψ(r), as a function of R¯. Insets show two configurations of the biases, h, as a function of the neuron preferred positions. (E) MSE of the stimulus estimate, obtained as the MAP (blue curve, scale on the left y-axis), or from samples (orange curve, scale on the right y-axis), as a function of R¯. Inset: MSE (sampling) as a function of the average tuning width. (PDF) [file pcbi.1012240.s003.pdf]

**A**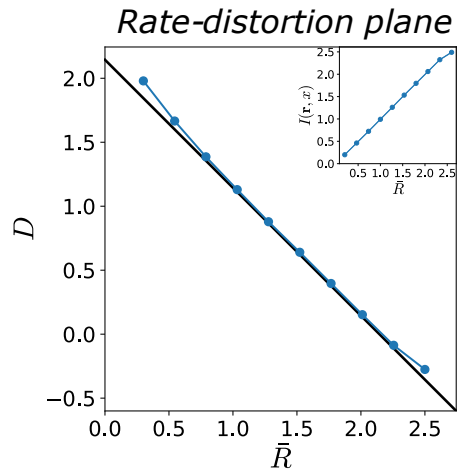**C**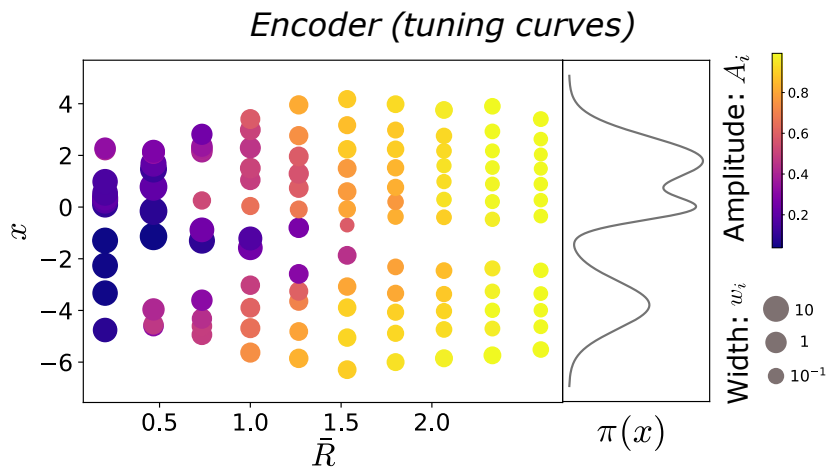**B**

*Stimulus vs generative dist.*

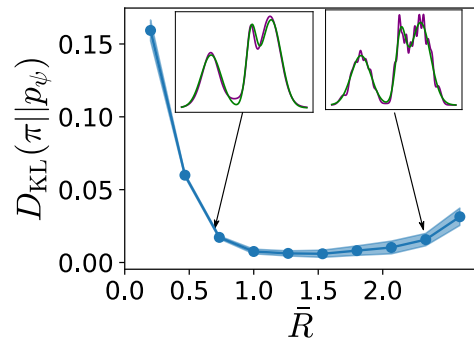**D**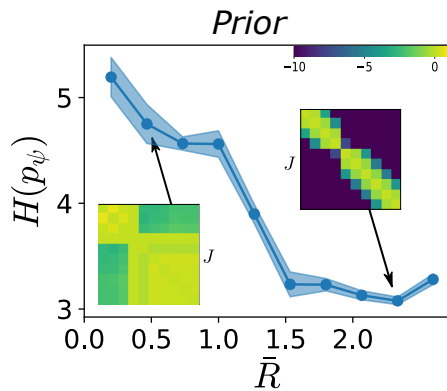**E**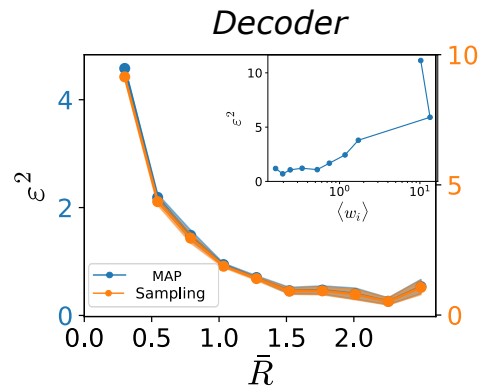

Supplement: S4 Fig — Same as Fig 4, but with π(x) a multimodal distribution: a mixture of three Gaussians with means {−4, 0, 2}; variances {1, 0.5, 1}; and mixture coefficients {0.3, 0.2, 0.5}. The decoder is Gaussian. (A) Solutions of the ELBO optimization problem as a function of target rate, D(R¯) (blue curve), and theoretical optimum, D = H(π) − R (black curve), in the rate-distortion plane. Values of R¯ where the solutions coincide with the theoretical optimum (grey region). Solutions depart from the optimal line when the rate is very low (poor generative model) or very high (saturated distortion). Inset: mutual information between stimuli and neural responses as a function of R¯. (B) DKL divergence between the stimulus and the generative distributions, as a function of R¯. Insets: two examples of comparison between stimulus (green curve) and generative distribution (purple curve). (C) Optimal tuning curves for different values of R¯. Each dot represents a neuron: the position on the y-axis corresponds to its preferred stimulus, the size of the dot is proportional to the tuning width, and the color refers to the amplitude (see legend). The curve on the right illustrates the data distribution, π(x). (D) Entropy of the prior distribution over neural activity, pψ(r), as a function of R¯. Insets show two configurations of the coupling matrices, with rows ordered according to the neurons’ preferred stimuli, and coupling strengths colored according to the legend. (E) MSE of the stimulus estimate, obtained as the MAP (blue curve, scale on the left y-axis), or from samples (orange curve, scale on the right y-axis), as a function of R¯. Inset: MSE (sampling) as a function of the average tuning width. (PDF) [file pcbi.1012240.s004.pdf]

**A***Training loss*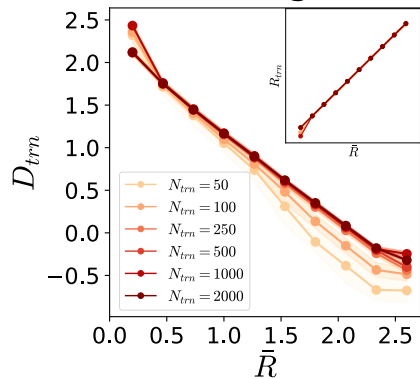**B***Stimulus vs generative dist.*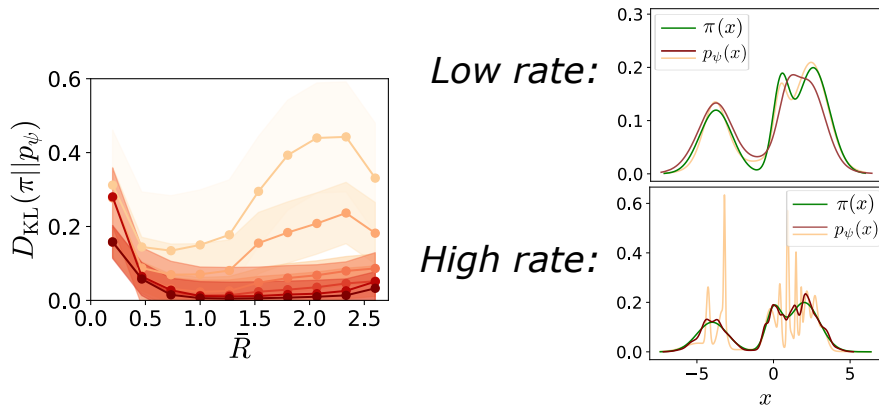**C***Encoder*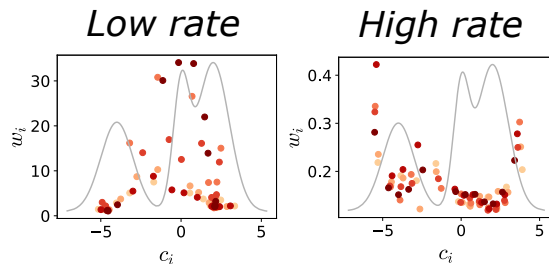**D***Decoder*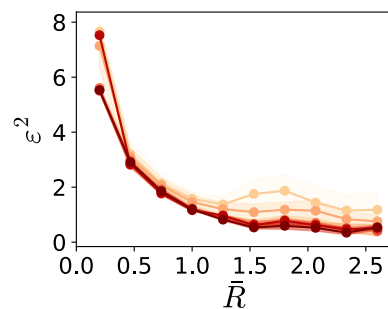*Test loss*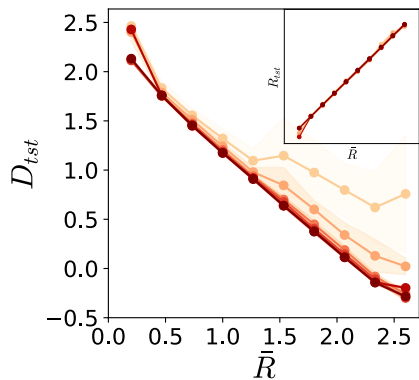

Supplement: S5 Fig — Same as Fig 6, but with π(x) a multimodal distribution: a mixture of three Gaussians with means {−4, 0, 2}; variances {1, 0.5, 1}; and mixture coefficients {0.3, 0.2, 0.5}. The legend in panel A serves as a legend for all panels. (A) Solutions of the ELBO optimization problem as functions of the target rate, for the training set (top) and for the test set (bottom). Top: distortion, Dtrn(R¯), and rate, Rtrn(R¯) (inset), for the training set as a function of the target rate, for different sizes of the training set, colored according to the legend. For smaller training sets, at higher rates the model tends to overfit the data, resulting in a lower training distortion than optimal (red line, large training set, same data as in S4 Fig). Bottom: distortion, Dtst(R¯), and rate, Rtst(R¯) (inset), for the test set as functions of the target rate, for different sizes of the training set. For smaller training sets, at higher rates the model does not generalize to unseen samples, resulting in a large distortion. (B) Left: Kullback-Leibler divergence between the stimulus and the generative distributions, as a function of R¯, for different sizes of the training set. At higher rates, the generative model fits poorly the stimulus distribution. Right: examples of comparisons between stimulus (green line) and generative distribution (red and orange line) at low (top) and high (bottom) rates, for different sizes of the training set, Ntrn = 100 and Ntrn = 2000, colored according to the legend as in panel A. (C) Tuning width, wi, as a function of the location of a preferred stimulus, ci (dots), at low (left) and high (right) rates, for different sizes of the training set, Ntrn = 100 and Ntrn = 2000. The grey curve represents the stimulus distribution, π(x). (D) MSE in the stimulus estimate, obtained as the MAP, as a function of R¯, for different sizes of the training set. (PDF) [file pcbi.1012240.s005.pdf]

$$\bar{R} = 1.0$$

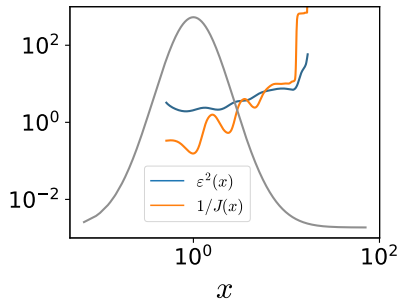

$$\bar{R} = 1.8$$

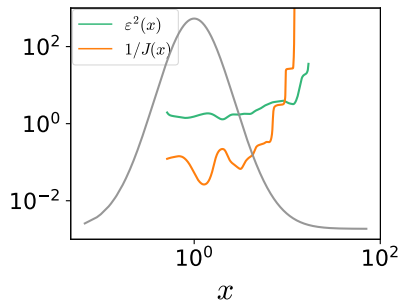

$$\bar{R} = 2.6$$

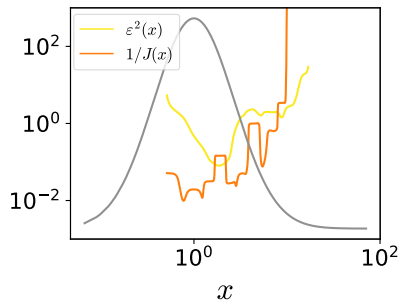

Supplement: S6 Fig — MSE (MAP estimate) (blue, green and yellow curves), and Cramer-Rao bound (inverse of the Fisher information, orange curves), as in Eq (19) as a function of x. (PDF) [file pcbi.1012240.s006.pdf]

**A***Neural density*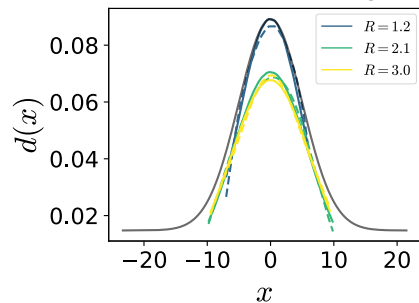**B***Tuning width*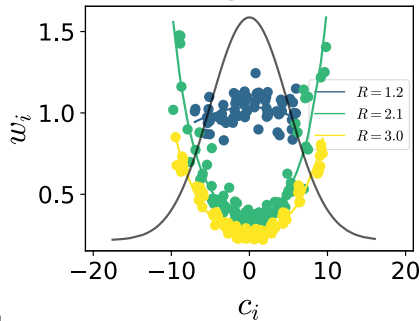**C***Density vs width*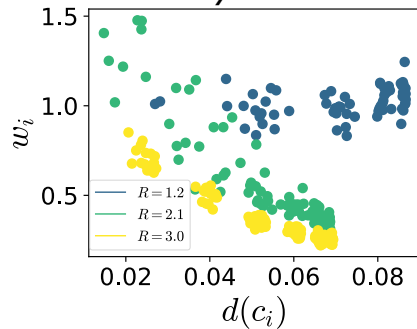**D***Error*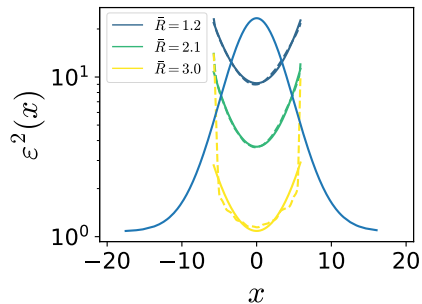**E***Error vs density*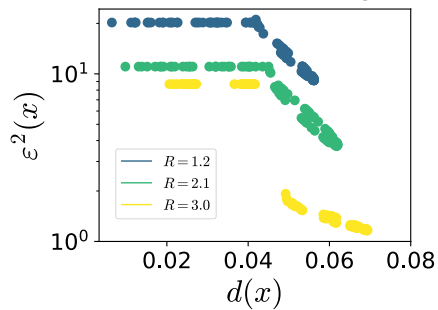**F***Error vs width*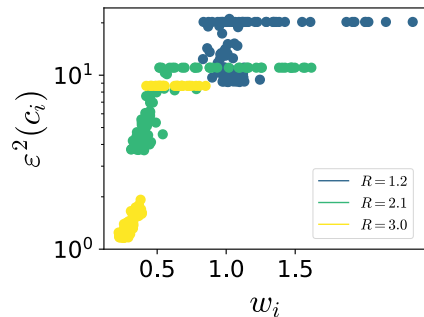

Supplement: S7 Fig — Same as Figs 7 and 8, in the case of a Gaussian distribution π(x)=N(0,5) (same of Fig 3) and Gaussian decoder. (A) Neural density as a function x (dashed curves) and power-law fits (solid curves, R2 = (0.96, 0.99, 0.99), γd = (0.99, 0.71, 0.64)), for three values of R¯ (low, intermediate, and high); the grey curve illustrates the stimulus distribution. The density is computed by applying kernel density estimation to the set of the preferred positions of the neurons. (B) Tuning width, wi, as a function of preferred stimuli, ci (dots), and power-law fits (solid curves, R2 = (0.09, 0.87, 0.92), γw = (−, 0.77, 0.66)) for three values of R¯; the grey curve illustrates the stimulus distribution. (C) Tuning width, wi, as a function of the neural density, d(ci), for three values of R¯; Pearson correlation coefficient ρ = (0.30, −0.91, −0.97). (D) MSE (estimate obtained through sampling) as a function of x (dashed curves), and power-law fits (solid curves, R2 = (0.99, 0.98, 0.62), γe = (1.37, 1.73, 1.86)), for three values of R¯. (E),(F) MSE as a function of the neural density (E) and tuning width (F), for three values of R¯; Pearson correlation coefficient ρdensity = (−0.84, −0.90, −0.91), ρwidth = (0.38, 0.79, 0.90). (PDF) [file pcbi.1012240.s007.pdf]

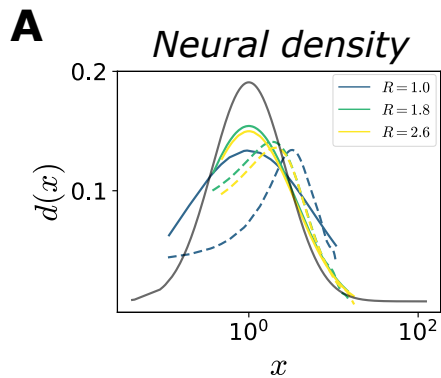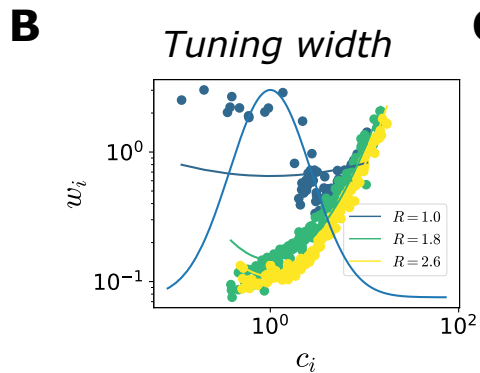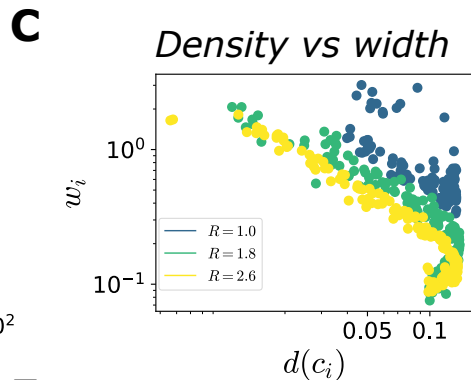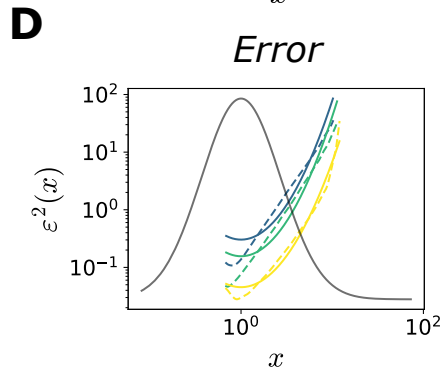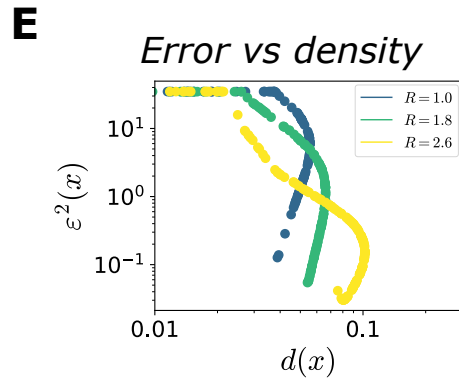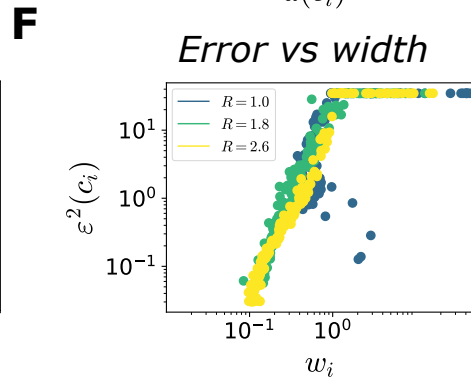

Supplement: S8 Fig — Same as Figs 7 and 8, in the case of log-normal decoder. (A) Neural density as a function x (dashed curves) and power-law fits (solid curves, R2 = (0.48, 0.94, 0.94), γd = (−, 0.60, 0.63)), for three values of R¯ (low, intermediate, and high); the grey curve illustrates the stimulus distribution. The density is computed by applying kernel density estimation to the set of the preferred positions of the neurons. (B) Tuning width, wi, as a function of preferred stimuli, ci (dots), and power-law fits (solid curves, R2 = (0.02, 0.90, 0.98), γw = (−, 0.74, 0.74)) for three values of R¯; the grey curve illustrates the stimulus distribution. (C) Tuning width, wi, as a function of the neural density, d(ci), for three values of R¯; Pearson correlation coefficient ρ = (−0.60, −0.87, −0.87). (D) MSE (MAP estimate) as a function of x (dashed curves), and power-law fits (solid curves, R2 = (0.90, 0.91, 0.97), γe = (2.09, 2.10, 1.89)), for three values of R¯. (E),(F) MSE as a function of the neural density (E) and tuning width (F), for three values of R¯; Pearson correlation coefficient ρdensity = (−0.86, −0.94, −0.86), ρwidth = (0.39, 0.29, 0.66). (PDF) [file pcbi.1012240.s008.pdf]

**A**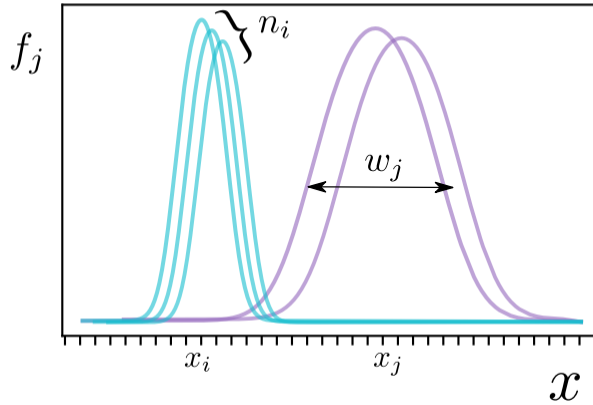**B**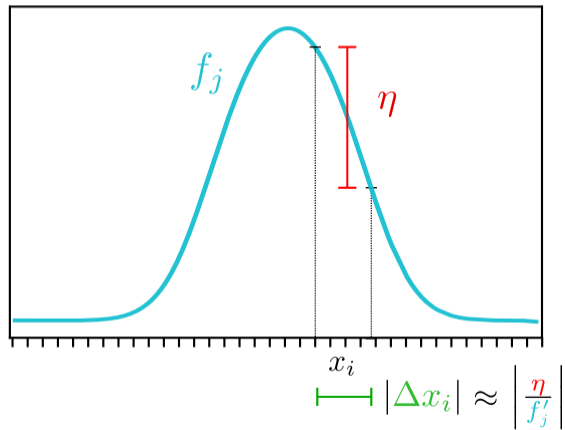

Supplement: S9 Fig — (A) A one-dimensional stimulus is encoded through bell-shaped tuning curves. The number of neurons whose preferred positions are a given stimulus, xi, is denoted by ni, while wi denotes the tuning width. (B) Approximate scaling of the error in stimulus estimate, Δxi, when the response of a neuron, with mean fj, is affected by a noise of standard deviation η. (PDF) [file pcbi.1012240.s009.pdf]
