# Supplementary material for: Jointly efficient encoding and decoding in neural populations
Source: PLoS Comput Biol. 2024 Jul 10;20(7):e1012240. doi: 10.1371/journal.pcbi.1012240 (PMC11262701; doi:10.1371/journal.pcbi.1012240)
Supplement: S1 Appendix — Demonstrations of the 3 properties of the solutions to the minimax problem of the Lagrangian, Eq (22), we listed in Materials and methods. (PDF) [file pcbi.1012240.s010.pdf]

# Jointly efficient encoding and decoding in neural populations

Simone Blanco Malerba[1,2], Aurora Micheli[1,†], Michael Woodford[3], and Rava Azeredo da Silveira[1,4,5]

**1** Laboratoire de Physique de l'Ecole Normale Supérieure, ENS, Université PSL, CNRS,3 Sorbonne Université, Université de Paris, Paris, France
**2** Institute for Neural Information Processing, Center for Molecular Neurobiology, University Medical Center Hamburg-Eppendorf, Hamburg, Germany
**3** Department of Economics, Columbia University, New York, United States of America
**4** Institute of Molecular and Clinical Ophthalmology Basel, Basel, Switzerland
**5** Faculty of Science, University of Basel, Basel, Switzerland
**†** Present address: Delft University of Technology, Delft, the Netherlands

## Supporting information

### S1 Appendix. Properties of the solutions to the minimax problem and maximization of the ELBO

Here, we demonstrate the 3 properties of the solutions to the minimax problem of the Lagrangian, Eq. (22) of the main text,

$$\min_{\{\theta,\psi\}} \max_{\beta\geq 0} \left\{ L(\theta,\psi,\beta) = D + \beta(R - \bar{R}) \right\}, \tag{S1}$$

we listed in Materials and methods. In particular, under which conditions the solutions we obtain maximize the ELBO. If the Lagrangian function is convex in the parameters $\{\theta, \psi\}$, then the alternated stochastic gradient descent-ascent algorithm converges to a saddle point [1], i.e., we have

$$L(\theta^*, \psi^*, \beta) \leq L(\theta^*, \psi^*, \beta^*) \leq L(\theta, \psi, \beta^*), \tag{S2}$$

for all feasible parameters and $\beta \geq 0$. According to the saddle point theorem (see, e.g., [2]), Eq. (S2) implies that $\{\theta^*, \psi^*\}$ is a solution of the problem defined in Eq. (20) of the main text (property 1),

$$\begin{aligned} \min_{\{\theta,\psi\}} \quad & D \\ \text{subject to} \quad & R \leq \bar{R}. \end{aligned} \tag{S3}$$

The convergence properties in the general case with $L$ possibly non convex in the parameters, but concave in $\beta$, are the object of ongoing research; Ref. [3] shows that a gradient descent-ascent algorithm converges to a stationary point of the function $g(\cdot) = \max_{\beta\geq 0} L(\cdot, \beta)$.

Regarding property 2, solutions of Eq. (S1) obey $\beta^*(R(\theta^*, \psi^*) - \bar{R}) = 0$, i.e., if $\beta^* > 0$, the constraint on the rate is satisfied as an equality, $R = \bar{R}$ (this mechanisms is also known as the complementarity slackness in the Karush–Kuhn–Tucker conditions [4]). Moreover, if the solution is a differentiable point and a saddle point (or,

more generally, a stationary point) of the Lagrangian, we have that $\frac{dD}{d\bar{R}}\Big|_{\theta^*,\psi^*} = -\beta^*$. (This can be shown by noting that

$$\frac{dL}{d\bar{R}}\bigg|_{\theta^*,\psi^*} = \frac{dD}{d\bar{R}}\bigg|_{\theta^*,\psi^*}, \tag{S4}$$

and that

$$\begin{aligned}\frac{dL}{d\bar{R}}\bigg|_{\theta^*,\psi^*,\beta^*} &= \frac{\partial\theta}{\partial\bar{R}}\frac{\partial L}{\partial\theta}\bigg|_{\theta^*,\psi^*,\beta^*} + \frac{\partial\psi}{\partial\bar{R}}\frac{\partial L}{\partial\psi}\bigg|_{\theta^*,\psi^*,\beta^*} + \frac{\partial\beta}{\partial\bar{R}}\frac{\partial L}{\partial\beta}\bigg|_{\theta^*,\psi^*,\beta^*} + \frac{\partial L}{\partial\bar{R}}\bigg|_{\theta^*,\psi^*,\beta^*}\\ &= -\beta^*,\end{aligned} \tag{S5}$$

since the partial derivatives evaluated at the stationary points vanish.)

Finally, regarding property 3, if the stationarity condition is satisfied and we find $\beta^* = 1$ as a result of our optimization scheme, then it is possible to show, under some assumptions, that the parameters $\{\theta^*, \psi^*\}$ maximize the ELBO. This is obviously true if the solution belongs to the line $D = H - R$, where the ELBO achieves its upper bound. In general, $\beta^* = 1$ implies that the ELBO is optimized if the distortion-rate curve, $D(\bar{R})$ (i.e., the curve defined by the solutions of Eq. (S3) as a function of $\bar{R}$), is convex. This observation can be proved with a simple geometric argument. We denote by $\bar{R}_1$ the point at which we have $dD/d\bar{R} = -1$; at this point, the tangent line to the distortion-rate curve is defined by $D = -\mathrm{ELBO}_1 - \bar{R}$ , with $-\mathrm{ELBO}_1 = D(\bar{R}_1) + \bar{R}_1$, as the constraint is satisfied as an equality, $R(\bar{R}_1) = \bar{R}_1$. The convexity of the distortion-rate function implies that it lies above this tangent line. Indeed, the convexity property implies that

$$D(\lambda\bar{R}_1 + (1-\lambda)\bar{R}_2) \leq \lambda D(\bar{R}_1) + (1-\lambda)D(\bar{R}_2), \tag{S6}$$

with $0 < \lambda < 1$. We now assume, without loss of generality, $\bar{R}_1 < \bar{R}_2$. By subtracting $D(\bar{R}_1)$ and dividing both sides in Eq. (S6) by $(1-\lambda)(\bar{R}_2 - \bar{R}_1) < 0$, we obtain

$$\frac{D\left(\lambda\bar{R}_1 + (1-\lambda)\bar{R}_2\right) - D(\bar{R}_1)}{\left(\lambda\bar{R}_1 + (1-\lambda)\bar{R}_2\right) - \bar{R}_1} \geq \frac{(1-\lambda)\left(D(\bar{R}_2) - D(\bar{R}_1)\right)}{(1-\lambda)(\bar{R}_2 - \bar{R}_1)}; \tag{S7}$$

we now take the limit $\lambda \to 1$, which yields

$$\frac{dD}{d\bar{R}}\bigg|_{\bar{R}_1} = -\beta^*(\bar{R}_1) \geq \frac{D(\bar{R}_2) - D(\bar{R}_1)}{\bar{R}_2 - \bar{R}_1}. \tag{S8}$$

Finally, by rearranging the terms, we obtain

$$D(\bar{R}_1) + \bar{R}_1 = -\mathrm{ELBO}_1 \leq D(\bar{R}_2) + \bar{R}_2, \tag{S9}$$

where we have used $\beta^*(\bar{R}_1) = 1$. We now define the negative ELBO at $\bar{R}_2$, $-\mathrm{ELBO}_2 = D(\bar{R}_2) + R(\bar{R}_2)$. Equation (S9) directly implies that $\mathrm{ELBO}_1 \geq \mathrm{ELBO}_2$ when the constraint is satisfied as an equality, $R(\bar{R}_2) = \bar{R}_2$. Instead, when $R(\bar{R}_2) < \bar{R}_2$, we can consider the problem defined in Eq. (S3) with $\bar{R}_3 = R(\bar{R}_2)$. In this case, we have $D(\bar{R}_2) = D(\bar{R}_3)$ and, since $D(\bar{R}_2)$ is achieved when $R = \bar{R}_3$, the constraint is satisfied as an equality, $R(\bar{R}_3) = \bar{R}_3$; thus, Eq. (S9) implies $\mathrm{ELBO}_1 \geq \mathrm{ELBO}_2 = \mathrm{ELBO}_3$. This proves that $\bar{R}_1$ maximizes the ELBO.

## References

1. Nedić A, Ozdaglar A. Subgradient methods for saddle-point problems. Journal of Optimization Theory and Applications. 2009;142(1):205–228. doi:10.1007/s10957-009-9522-7.

2. Boyd S, Boyd SP, Vandenberghe L. Convex optimization. Cambridge university press; 2004.

3. Lin T, Jin C, Jordan MI. On gradient descent ascent for nonconvex-concave minimax problems. International Conference on Machine Learning. 2020;37. doi:https://doi.org/10.48550/arXiv.1906.00331.

4. Arrow KJ, Azawa H, Hurwicz L, Uzawa H, Chenery HB, Johnson SM, et al. Studies in linear and non-linear programming. vol. 2. Stanford University Press; 1958.
