# Supplementary material for: Jointly efficient encoding and decoding in neural populations
Source: PLoS Comput Biol. 2024 Jul 10;20(7):e1012240. doi: 10.1371/journal.pcbi.1012240 (PMC11262701; doi:10.1371/journal.pcbi.1012240)
Supplement: S2 Appendix — (PDF) [file pcbi.1012240.s011.pdf]

Jointly efficient encoding and decoding in neural populations

Simone Blanco Malerba[1,2], Aurora Micheli[1,†], Michael Woodford[3], and Rava Azeredo da Silveira[1,4,5]

1 Laboratoire de Physique de l'Ecole Normale Supérieure, ENS, Université PSL, CNRS,3 Sorbonne Université, Université de Paris, Paris, France
2 Institute for Neural Information Processing, Center for Molecular Neurobiology, University Medical Center Hamburg-Eppendorf, Hamburg, Germany
3 Department of Economics, Columbia University, New York, United States of America
4 Institute of Molecular and Clinical Ophthalmology Basel, Basel, Switzerland
5 Faculty of Science, University of Basel, Basel, Switzerland
† Present address: Delft University of Technology, Delft, the Netherlands

Supporting information

S2 Appendix. Numerical approaches in the case of large neural populations

To extend our model to larger populations, there are two numerical issues to consider. The first one concerns the distortion term and the gradient with respect to the parameters of the encoder. In order to obtain a low-variance estimate of the gradient, an approach is to use the so-called reparametrization trick together with a continuous relaxation of the discrete random variable, $\mathbf{r}$, (or Gumbel-softmax trick [1, 2]), and calculate the gradient as

$$\begin{aligned} \nabla_\theta D(x) &= \nabla_\theta \left\langle \log p_\psi(x|\mathbf{r}) \right\rangle_{q_\theta(\mathbf{r}|x)} \\ &\approx \left\langle \nabla_\theta \log p_\psi(x|f_\theta(\xi, x)) \right\rangle_{p(\xi)} , \end{aligned} \tag{S1}$$

with $p(\xi) = \mathcal{U}(0, 1)$. Here,

$$f_\theta(\xi, x)) = \mathcal{S}\left(\frac{\boldsymbol{\eta}_\theta(x) + \mathcal{S}^{-1}(\xi)}{\tau}\right) \tag{S2}$$

depends deterministically on the parameters θ through the natural parameters of the encoder; the hyperparameter τ controls the steepness of the logistic function, and consequently the bias-variance trade-off for the gradient; simulations with values of $\tau = 10^{-2}$ yield results comparable to the ones presented here.

The second issue pertains to the form of the rate. Its expression can be simplified as

$$\begin{aligned} D_{\mathrm{KL}}\left(q_\theta(\mathbf{r}|x)||p_\psi(\mathbf{r})\right) &= \left\langle \left(\boldsymbol{\eta}(x) - \mathbf{h}\right)\mathbf{r} - \mathbf{r}^T J \mathbf{r} \right\rangle_{q_\theta(\mathbf{r}|x)} - \sum_{i=1}^{N} \log\left(1 + e^{\eta_i(x)}\right) + \log Z \\ &= \left(\boldsymbol{\eta}(x) - \mathbf{h}\right)\mathbf{p}(x) - \mathbf{p}^T(x) J \mathbf{p}(x) - \sum_{i=1}^{N} \log\left(1 + e^{\eta_i(x)}\right) + \log Z, \end{aligned} \tag{S3}$$

where $\mathbf{p}(x) = \mathcal{S}(\boldsymbol{\eta}(x))$ is the vector of mean parameters of the encoding distribution (i.e., the spiking probability of neurons). In the expectation of the quadratic form, $\langle \mathbf{r}^T J \mathbf{r} \rangle_{q_\theta(\mathbf{r}|x)} = \mathrm{tr}(K_{\mathbf{rr}} J) + \mathbf{p}^T(x) J \mathbf{p}(x)$, we have that $\mathrm{tr}(K_{\mathbf{rr}} J) = 0$, as the covariance matrix of the activity patterns, $K_{\mathbf{rr}}$, is proportional to the identity, and the diagonal elements of J vanish. Here, the numerical load is in computing the gradient of the log-partition function, $\log Z$; this can be done by Monte Carlo methods [3].

References

1. Maddison CJ, Mnih A, Teh YW. The Concrete Distribution: A Continuous Relaxation of Discrete Random Variables. arXiv preprint arXiv:161100712. 2016;.

2. Jang E, Gu S, Poole B. Categorical reparameterization with gumbel-softmax. arXiv preprint arXiv:161101144. 2016;.

3. Rolfe JT. Discrete Variational Autoencoders. arXiv preprint arXiv:160902200. 2016;doi:https://doi.org/10.48550/arXiv.1609.02200.
