# Supplementary material for: Jointly efficient encoding and decoding in neural populations
Source: PLoS Comput Biol. 2024 Jul 10;20(7):e1012240. doi: 10.1371/journal.pcbi.1012240 (PMC11262701; doi:10.1371/journal.pcbi.1012240)
Supplement: S3 Appendix — We provide an alternative derivation, based on scaling arguments, of the results in Ref. [10]. (PDF) [file pcbi.1012240.s012.pdf]

Jointly efficient encoding and decoding in neural populations

Simone Blanco Malerba[1,2], Aurora Micheli[1,†], Michael Woodford[3], and Rava Azeredo da Silveira[1,4,5]

1 Laboratoire de Physique de l'Ecole Normale Supérieure, ENS, Université PSL, CNRS,3 Sorbonne Université, Université de Paris, Paris, France
2 Institute for Neural Information Processing, Center for Molecular Neurobiology, University Medical Center Hamburg-Eppendorf, Hamburg, Germany
3 Department of Economics, Columbia University, New York, United States of America
4 Institute of Molecular and Clinical Ophthalmology Basel, Basel, Switzerland
5 Faculty of Science, University of Basel, Basel, Switzerland
† Present address: Delft University of Technology, Delft, the Netherlands

Supporting information

S3 Appendix. Optimally heterogeneous allocation of neural resources

We provide an alternative derivation, based on scaling arguments, of the results in Ref. [1]. We consider a population of N neurons, in which neuron i responds to a continuous scalar stimulus, x, according to a bell-shaped tuning curve, $f_i(x)$. We consider a discretization of the stimulus space, $x = \{x_i\}_{i=1}^{L}$, and we denote by d_i the number of neurons whose preferred stimulus is x_i and by w_i their tuning width (Fig S9A). The number of neurons encoding information about stimulus x_i scales as

$$\#\text{neurons} = M_i \sim d_i w_i, \tag{S1}$$

as increasing the number of neurons and the tuning width (both of which, we assume, vary sufficiently smoothly with position) each increases the number of neurons that 'monitor' a given of the stimulus. We assume that neural responses, r, are corrupted by noise with standard deviation η. Through a simple geometric argument (Fig S9B), we estimate the square of the difference between the stimulus estimate based on the activity of neuron j and the true stimulus, i.e., the squared error, as

$$(\hat{x}_i - x_i)^2 \equiv \Delta x_i^2 \approx \left(\frac{\eta}{f_j'(x_i)} \right)^2, \tag{S2}$$

where $f_j'(x)$ denotes the slope of the tuning curve j at x_i. The derivative of a bell-shaped tuning curve scales as $f_i'(x) \sim f_i(x)/w_i$; if noise has a Poisson distribution, the variance of the response is equal to the mean, so that Eq. (S2) can be written as

$$\Delta x_i^2 \sim \left(\frac{\text{const}}{w_i} \right)^{-2} \sim w_i^2. \tag{S3}$$

As M independent neurons encode stimulus x_i, we can average the single estimates from each of the neuron to obtain a more faithful estimate. The variance of this

population estimate, i.e., the MSE, for stimulus i, scales as

$$\begin{aligned}\varepsilon_i^2 = \mathrm{Var}\left(\frac{1}{M_i}\sum_{j=1}^{M}(\Delta x_i)_j\right) &= \frac{1}{M^2}\sum_{j=1}^{M_i}(\Delta x_i^2)_j \\ &\approx \frac{w_i^2}{M_i} \\ &\approx \frac{w_i}{d_i},\end{aligned} \tag{S4}$$

where the last equality follows from Eq. (S1). By taking the limit of an infinitely fine discretization, $L \to \infty$, and assuming that the population size is large enough so that the quantities d_i and w_i vary smoothly, we can consider a continuum limit with

$$d_i \to d(x), \tag{S5}$$

the neural density,

$$w_i \to w(x), \tag{S6}$$

the tuning width, and

$$M_i \to M(x) = d(x)w(x) \tag{S7}$$

We will require an additional constraint to find optimal solutions. Different forms of constraint can be imposed. The constraint that reproduces the results of [1] ensures a 'uniform coverage' across stimuli, i.e., $M(x) = \text{constant}$, or

$$w(x) \sim \frac{1}{d(x)}. \tag{S8}$$

The efficient coding hypothesis posits that neurons are arranged so as to maximize the mutual information between stimuli and neural responses. An approximation of the mutual information in terms of the Fisher information, $J(x)$, in the asymptotic limit, can be obtained as

$$I(r, x) = \int dx \pi(x) \log\left(J(x)\right) + \text{const}, \tag{S9}$$

where $\pi(x)$ is the distribution of stimuli and const denotes terms that don't depend on the neural responses [2]. The Fisher information is a lower bound to the variance of any unbiased estimator; if we assume that the bound is tight, we have that

$$J(x) \approx \frac{1}{\varepsilon^2(x)} \sim d(x)^2, \tag{S10}$$

where $\varepsilon^2(x)$ corresponds to the continuum limit of Eq. (S4) and we used the scaling relation of Eq. (S8).

We now maximize the mutual information subject to a constraint on the neural resources–here, merely, the number of neurons–by optimizing the sum of the two terms

$$\max_{d(x)}\left\{\int dx \pi(x) \log\left(d(x)^2\right) + \beta \int dx d(x)\right\}. \tag{S11}$$

By taking a functional derivative with respect to $d(x)$ and setting it to zero, we obtain

$$d(x) \sim \pi(x), \tag{S12}$$

and, consequently, the scaling of the MSE as

$$\varepsilon^2(x) \sim \frac{1}{p^2(x)}. \tag{S13}$$

We note that Eq. (S11) can be rewritten in terms of the Fisher information, as

$$\max_{J(x)} \left\{ \int dx \pi(x) \log J(x) + \beta \int dx \sqrt{J(x)} \right\}. \tag{S14}$$

Equation (S14) matches the objective proposed in Refs. [3, 4], and yield an optimal allocation of coding resources with $J(x) \propto p(x)^2$. This solution can be generalized by modifying the two exponents of $J(x)$ in Eq. (S14) [5, 6].

Main differences with our model. Our model is similar to the one presented above, but it differs from it in ways which complicate analytical calculations and give rise to more complex behaviors.

- The first difference is in the noise model: we assume binary neurons, while the above calculations are carried out with Poisson neurons, an assumption which allows the simplification in Eq. (S3).
- The second difference is that, in our formulation, the tuning width and neural density are free to vary independently, while here the optimization is conducted over a single scalar function, $w(x)$. As a result, we can achieve a non-uniform coverage across stimuli. The rate term controls the width of tuning curves, and broad tuning curves emerge without further constraints.
- The third difference is that we assume a finite population size, rather than he asymptotic $N \to \infty$ limit.
- Our loss function is similar to that in Eq. (S11) for what concerns the first term, which represents the mutual information between stimuli and neural responses (although in our case we have a lower bound, which depends also on the decoder), but the constraint is more intricate due to its dependence on the generative model.
- Finally, our model does not require the full knowledge of the prior distribution over stimuli; solutions are derived on the basis of data samples.

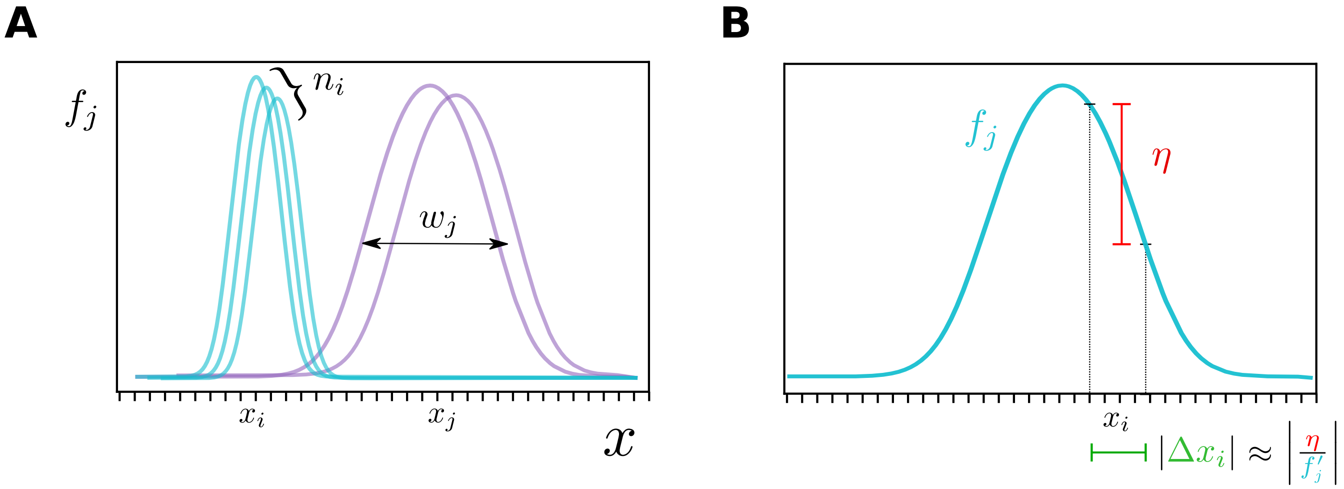

Fig S9. Population coding model with bell-shaped tuning curves. (**A**) A one-dimensional stimulus is encoded through bell-shaped tuning curves. The number of neurons whose preferred positions are a given stimulus, x_i, is denoted by n_i, while w_i denotes the tuning width. (**B**) Approximate scaling of the error in stimulus estimate, Δx_i, when the response of a neuron, with mean f_j, is affected by a noise of standard deviation η.

References

1. Ganguli D, Simoncelli EP. Efficient Sensory Encoding and Bayesian Inference with Heterogeneous Neural Populations. Neural Computation. 2014;26(10):2103–2134. doi:10.1162/NECO_a_00638.

2. Brunel N, Nadal JP. Mutual Information, Fisher Information, and Population Coding. Neural Computation. 1998;10(7):1731–1757. doi:10.1162/089976698300017115.

3. Wei XX, Stocker AA. Efficient coding provides a direct link between prior and likelihood in perceptual Bayesian inference. Advances in Neural Information Processing Systems. 2012;25:1304–1312.

4. Wei XX, Stocker AA. Bayesian inference with efficient neural population codes. In: Lecture Notes in Computer Science (including subseries Lecture Notes in Artificial Intelligence and Lecture Notes in Bioinformatics). vol. 7552 LNCS; 2012. p. 523–530.

5. Morais MJ, Pillow JW. Power-law efficient neural codes provide general link between perceptual bias and discriminability. Advances in Neural Information Processing Systems. 2018;31:5071–5080.

6. Prat-Carrabin A, Woodford M. Bias and variance of the Bayesian-mean decoder. Advances in Neural Information Processing Systems. 2021;34:23793–23805.
