# Supplementary material for: Jointly efficient encoding and decoding in neural populations
Source: PLoS Comput Biol. 2024 Jul 10;20(7):e1012240. doi: 10.1371/journal.pcbi.1012240 (PMC11262701; doi:10.1371/journal.pcbi.1012240)
Supplement: Attachment — Submitted filename: PLoS Comp Bio Review.pdf [file pcbi.1012240.s013.pdf]

Dear Editor,

Thank you for the opportunity to review this manuscript. In this work, the authors conducted studies of optimal neural code using the techniques of varitional autoencoder (VAE). The authors established connections of their approach to the classical efficient coding literature through the rate-distortion interpretation of VAEs. The authors were able to relate their results with previous literature on similar questions but using a more analytical approach, and also offered comparison of their model with empirical data.

Overall, I thought this article is an interesting and novel application of VAE to neuroscience, and can potentially be applied to many other questions in similar domain. I do have some comments/concerns as outlined below, but they do not distract my overall positive impression of the work.

Main Questions

1. The results in Fig. 6 - 8 focus on the properties of neural coding after training the VAE, and compare them to those from other efficient coding literature. However, I am concerned about the potential impact of the choice of the generative model $q_{\phi}(x|r)$ on the encoding, and whether results obtained under one particular $q_{\phi}(x|r)$ can meaningfully generalize to a different $q'_{\phi}(x|r)$. For example, will the fitted exponent γ_e in Fig. 7 differ if we use log-normal as the base distribution in the decoder instead? More boardly, in a VAE framework, can we meaningful isolate the impact of constraints (i.e., rate, stimulus distrution) on encoder, without considering the limit of the generative model itself?

2. One potential strength of the VAE approach is it allows for optimization of more complex aspect of the neural codes. In the current manuscript however, only location and width of the tuning curves are allowed to change. Can the shape of the tuning curve itself be optimized within a general parametric family? This will allow for a much stronger test of theory, for example, does log-normal tuning curve (Nover, Anderson, DeAngelis 2005) naturally emerge when the model is applied to motion? The current result in Fig. 8 can also be obtained using simpler framework such as in Ganguli & Simoncelli 2016.

Other Comments:

3. There have been studies in the literature (e.g., Wang, Stocker & Lee 2016; Park & Pillow 2020; Schaffner et al., 2023) on optimal encoding for different loss function in the decoding. These results are quite relevant for the current paper and should be discussed. For example, the γ_e in Fig. 7 is closer to 1 presumably because the objective for VAE is closer to a L_2 loss rather than the standard infomax.

4. After training the VAE, since the encoding model $p(r|x)$ is directly accessible, it seems straightforward to compute Fisher information analytically, which will make comparisons to previous results and computing discrimination threshold (Fig. 8C) more direct, instead of relying on the proxy through MSE.

5. [Line 170] The connection between VAE and efficient coding through the rate-distortion interpretation is quite important for the paper. Some of the results discussed here should be moved to the results or the introduction section of the paper.

6. [Line 415] Should the reference be Fig. 4C instead of 4B?

7. [Line 574 - 604] I am comfortable with interpretation the rate quantity R as an abstract form of constraint, but not sure if it makes sense as “deviation from spontaneous activity”. As in Ref. 18 of the manuscript, spontaneous activity itself can be changed through learning and development, so it does not feel as fundamental as things such as metabolic constraint.
