# Supplementary material for: Jointly efficient encoding and decoding in neural populations
Source: PLoS Comput Biol. 2024 Jul 10;20(7):e1012240. doi: 10.1371/journal.pcbi.1012240 (PMC11262701; doi:10.1371/journal.pcbi.1012240)
Supplement: Attachment — Submitted filename: neuralVAE_ResponseToReviewers-resubmission.pdf [file pcbi.1012240.s014.pdf]

Response to the editor and reviewer reports, manuscript "Jointly efficient encoding and decoding in neural populations"

We thank the editor and the reviewers for their detailed comments on our work. We address all their comments below and in the revised manuscript. Hereafter, first we summarize the main changes and additions in the revised manuscript. Next, we provide detailed responses to the points raised by the editor and the reviewers; our responses appear in blue font. Wherever relevant, we point to corresponding additions or modifications brought to the manuscript; all the added or modified passages in the revised manuscript appear in pink font.

Overview of the major changes and additions brought to the manuscript

Here, we provide an overview of the changes made to the manuscript. These changes pertain to new results as well as to additions or modifications in the text. We have modified the text to clarify the purpose, structure, and conceptual novelty of the coding framework proposed in the paper. Following remarks from the editor and reviewer, we have conducted additional theoretical and numerical studies that address questions on the generalization properties of the model, the role of the population size, the impact of different assumptions on the functional forms of the model on the structure of results, and the comparison between the model and data. New material and major changes are listed as follows.

1) We have improved the exposition of the proposed coding framework. Specifically, we have reworked the description of the model and the different parts of the objective function (see sections "Generative Model (Decoder)," "Recognition Model (Encoder)," and "Training Objective" in Materials and methods). We have also entirely redone Fig. 1 for the sake of clarity and thoroughness. Following the suggestion of one of the reviewers, in the revised manuscript, we now introduce the generative model (decoder) before introducing the recognition model (encoder), as is customary in the machine learning literature. To comply with another reviewer who was favorable to our derivation of the VAE, we have rewritten the section "Training objective" and now provide three different derivations of the VAE objective function, including a derivation following the original derivation of Kingma and Welling; we believe that presenting these alternative views in a same section will be of benefit to readers from diverse backgrounds.

   In addition to restructuring and complementing the overall presentation of the model, we have also improved the exposition of the generative and recognition models, and we have clarified and motivated our assumptions (e.g., the assumption of binary neurons, and that of independent noise). For the reader familiar with other approaches based on generative models, we now emphasize the connections between our model and other generative models in neuroscience (e.g., the Helmoltz machine). We have also updated our notation to align with the literature on generative models. Finally, we have moved to the Appendix the technical passages previously included in the section "Constrained optimization and connection with efficient coding" of Materials and methods (Section S1 Appendix), where we now highlight the main steps and central results.

2) The revised manuscript features extensive discussions that compare our approach to similar ones in both the generative modeling literature and the efficient coding literature; we describe similarities, emphasized differences, and note the main points of novelty put forth in our framework. The main differences between our approach and classic efficient coding

approaches, as well as some of the new results from our approach, are as follows. First, in our model the terms quantifying the coding accuracy and the constraint on neural resources are both derived from the same loss function that reflects the requirement of maximizing the evidence. In classic efficient coding models, the choice of the functional forms of the two terms (referring to coding accuracy and cost respectively) is arbitrary to a large degree. Second, in our model the optimization is carried out in the absence of the full knowledge of the stimulus distribution: solutions are learned on the basis of a finite volume of data samples (observations). By contrast, classic models of efficient coding assume exact 'Bayesian inversion' (i.e., correct posteriors) which requires full knowledge of the stimulus distribution. Third, our approach yields broad neural tuning curve in the regime of intermediate values of the rate. In efficient coding, unless additional constraints be imposed, optimality demands arbitrarily narrow tuning curves (in the case of a large neural population). Forth, we show that the regime of intermediate rate is advantageous in that solutions (*i*) achieve low coding error together with a generative model that captures the stimulus distribution accurately, and (*ii*) generalize to unseen data.

In the revised manuscript, we discuss these points in the Abstract, in the Introduction (lines 51-54, 67-72), in the Results section "Optimal allocation of coding resources and coding performance" and the new section "Generalization and the role of the rate as regularizer," in the Discussion (new section "VAE, efficient coding, and learning"), and in the S3 Appendix.

3) We have expanded the Materials and methods section "Numerical optimization and related computations," to include details about parameters initialization and fitting procedures (lines 313-318 and 326-336).

4) We now discuss the interpretation of the VAE framework as an extended efficient coding approach, in the Results section (lines 346-351).

5) We have conducted a number of new numerical studies to examine the influence of the choice of functional forms in the model, and in particular the interplay of the functional forms assigned to the generative model and that of the stimulus distribution. As suggested by Reviewer 1, we now report and discuss results obtained using a log-normal decoder instead of a Gaussian decoder (Figs. S2 and S8). The new results are qualitatively similar to the ones obtained previously in the Gaussian case, but the generative model exhibits a better fit to heavy-tail distributions of the stimulus. An example of the latter is the distribution of acoustic frequencies, in the context of which we compare the predictions of our model to psychophysical observations. In the revised manuscript, we also investigate the role of the structure of the prior distribution of neural activity. In particular, we show that constraining the flexibility of this distribution (e.g., by replacing the Ising model by a product of Bernoulli distribution), limits the expressivity of the generative model, yielding a suboptimal fit of the stimulus distribution and less accurate coding (see Fig. S3).

6) We have investigated the impact of the population size on the properties of the solution, and we report results from this investigation in the revised manuscript (see Fig. 5 and lines 429-436).

7) We have included a new Results section, titled "Generalization and role of the rate as regularizer," together with two additional figures (Figs. 6 and S5), in the revised manuscript. The new material reflects new numerical investigations carried out to address

the question of generalization, following the suggestion of Reviewer 3. In brief, by carrying out the optimization for different volumes of training data, we extracted the way in which the constraint on the rate controls the generalization properties of the model. We found that intermediate values of the rate constraint allow for accurate coding while preventing overfitting.

8) We have corrected a numerical error in and improved the section “Case study: neural encoding of acoustic frequencies” (lines 600-633). There, we have also added new results (see Fig. 9); specifically, we now compare two choices of the decoder functional form, Gaussian and log-normal. We observe that the latter achieves a better performance in terms of ELBO, provides a superior fit to the heavy-tailed distribution of acoustic frequencies, and, importantly, yields a closer agreement with experimental psychophysical data.

Editor:

All reviewers find your work novel, well-written, and of general interest to the computational neuroscience community. However, we agree with reviewers R2, R3, and R4 that the form and purpose of the generative model in the proposed coding framework requires a more thorough explanation and clarification, as it is somewhat unconventional and thus potentially confusing.

We thank the editor for the generous take on our work and for this suggestion. We have now extensively rewritten parts of the manuscripts to clarify the exposition and motivations of our model. For a detailed response on this point, see points 1, 2, 5, 6, and 7 in the summary above and several of the responses to the reviewers, below.

In that context, R1 also raises the important question of what limitations the chosen Gaussian model imposes on generalizability.

Following the suggestion of Reviewer 1, we have now conducted a set of numerical investigations to address the impact of the choice of functional forms of the different elements of the model, and in particular the choices of the form of the decoding distribution and of the prior over neural activity; for details, see point 5 in the summary above and response to reviewer 1. We have now also carried out a detailed numerical study of the issue of generalization in our model. Our results are summarized in a new Results section, titled “Generalization and the role of the rate as regularizer,” included in the revised manuscript. For further comments on this, see point 7 in the summary above and several of the responses to the reviewers, below.

Furthermore, we echo the concerns (mainly by R2 and R3) that the manuscript would benefit from a more thorough discussion of the conceptual advances over previous related work, and its broader impact on our understanding of neural representations of sensory information in the brain.

We thank the editor and the reviewers for this suggestion. The revised manuscript now includes extensive discussions of the conceptual advances associated with the proposed framework, and well as detailed comments that compare and contrast the proposed approach to earlier models based on ideas of efficient coding or generative modeling. For a brief account, see point 2 in the summary above. We also discuss this in detail in our responses to the reviewers, below.

Finally, it is important that a revision resolves the concerns of R3 regarding a potentially incorrect model-to-data comparison.

We thank the reviewer for pointing out the discrepancy in the model-to-data comparison. We have now corrected it, and we have further incremented the corresponding section by including a report of new numerical investigations; see point 8 of the summary above and the response to Reviewer 3, below.

Reviewer 1:

The authors established connections of their approach to the classical efficient coding literature through the rate-distortion interpretation of VAEs. The authors were able to relate their results with previous literature on similar questions but using a more analytical approach, and also offered comparison of their model with empirical data. Overall, I thought this article is an interesting and novel application of VAE to neuroscience, and can potentially be applied to many other questions in similar domain. I do have some comments/concerns as outlined below, but they do not distract my overall positive impression of the work.

Main Questions

1. The results in Fig. 6 - 8 focus on the properties of neural coding after training the VAE, and compare them to those from other efficient coding literature. However, I am concerned about the potential impact of the choice of the generative model $q_{\phi}(x|r)$ on the encoding, and whether results obtained under one particular $q_{\phi}(x|r)$ can meaningfully generalize to a different $q'_{\phi}(x|r)$. For example, will the fitted exponent γ_e in Fig. 7 differ if we use log-normal as the base distribution in the decoder instead? More broadly, in a VAE framework, can we meaningful isolate the impact of constraints (i.e., rate, stimulus distribution) on encoder, without considering the limit of the generative model itself?

We thank the reviewer for encouraging us to return to the question of the respective roles of the various constraints imposed on the VAE model. It seems to us that this question is not only a deep one but also one that may not have been the object of a sufficiently systematic study. Part of the reason for this is that while the form of the solution of the VAE minimization prescription depends on the constraints imposed to the encoder and the decoder, it is difficult to classify the set of possible constraints and to map a classification to corresponding features of the solution. Quite generally, restrictions imposed on the decoder are necessary to obtaining non-trivial solutions. This is because a completely flexible generative model can match the prior conditional on any latent variable, $\mathbf{r}$, yielding a distortion equal to the negative entropy of the data, $-H(x)$. In turn, by choosing $p(r)$ to consist of a single atom, the rate can be set to zero. Once constraints are imposed, it is difficult to isolate the effect of the various restrictions: the minimization prescription couples the encoder and decoder, so that the various restrictions 'interact' in determining the form of the solution. For example, a simpler, less flexible decoder will induce the encoder to retain more information about the data in the neural activity.

In machine learning, constraints are to a great extent dictated by demands of tractability. In the context of neural systems, however, it is natural to think that the physiology of neurons imposes restrictions on the form of the permissible distributions. This was one of our motivations. Correspondingly, we assume an encoder that conforms to the observation of broad tuning curves in sensory neurons. Similarly, we assume that 'output neurons' in the decoder are also

relatively simple noisy units, while their mean activity and the magnitude of the noise can be flexibly selected (e.g., by a deep neural network). Thus, in our case, as opposed to the typical machine learning scenario, constraints are inspired by models of neurons.

As compared with classical formulations of efficient coding, our VAE approach differs in an important way, which is related to the presence of constraints on the encoder and decoder. Classical formulations of efficient coding assume noisy encoding of stimuli (e.g., in the activity of a population of noisy neurons) but allow for optimal decoding by 'Bayesian inversion.' By contrast, the restrictions imposed on the VAE architecture relax the assumption of perfect Bayesian inversion (indeed, the VAE can be seen as an implementation of variational Bayesian inversion). The notable advantage of this approach is that a solution can be learned (through minimization) based *only* on the available data, whereas perfect Bayesian inversion requires *knowledge of the full prior* over the data. The price paid for this benefit is the introduction of (arbitrary, even if motivated) constraints on the permissible encoder and decoder models. We had not made this point sufficiently explicitly in the previous version of our manuscript. In the revised version, we discuss this important conceptual point in the Abstract, in the Introduction (lines 51-54), and in the Discussion (lines 651-654 and 670-677).

This is why it seemed important to us to show, as we do in the manuscript, that without fine-tuning the constraints one can obtain solutions on the statistics of neural coding that match the solutions obtained in classical models of efficient coding. Specifically, we recover a power-law dependence of the allocation (over the stimulus space) of the coding performance, an inverse relation between tuning width and stimulus distribution, and a proportionate relation between neural density and stimulus distribution. Thus, the VAE approach we discuss in the manuscript, while more flexible than classic efficient coding models (in that it does not assume perfect Bayesian inversion), still predicts similar relations between the allocation of neural resources and the stimulus prior. Furthermore, and importantly, in efficient coding models the specific relations obtained depends on the (ad hoc) choice of the objective function and the cost; by contrast, the VAE is a model of unsupervised learning, and both terms in the optimization function (ELBO) result from the requirement of maximizing the evidence. We now discuss this second important conceptual distinction between the VAE approach and efficient coding approaches in lines 658-662.

In order to clarify the above points, we have included a discussion of the role of constraints on the behavior of the model in the revised manuscript (lines 407-413 and 536-539 of Results and 678-700 of Discussion). Furthermore, and more concretely, to illustrate these points in a quantitative manner, we have followed the reviewer's recommendation to explore the behavior of the model for a different form of the generative model, namely when it is assumed to be log-normal instead of Gaussian. As mentioned above, when the generative model matches the form of the prior over stimuli, the ELBO can be maximized to larger values, but at the cost of posterior collapse. We illustrate this in Fig. S2. We also examined the performance of the model in fitting data on auditory discriminations when using a log-normal form in the generative model. We observed that this choice provides a better fit to the human data, yielding a mean squared error with a stronger dependence on the stimulus distribution (as compared to a Gaussian generative model), with an exponent γ_e closer to 1 (see Fig. 9).

The modification addressed above (log-normal vs. Gaussian) concerns the conditional part of the generative model. It is also possible to consider alternative constraints on the marginal part of the generative model. In order to provide a thorough exploration of the role of constraints on the generative model, we also explored a more stringent constraint on the marginal

distribution over the neural activity. Specifically, we investigated the implications of assuming that the marginal distribution is independent; in other words, we set the coupling matrix, J, to zero. As a consequence, the marginal distribution over the neural activity becomes a product of Bernoulli distributions, each with a single parameter to train. This represents a much less flexible form of the generative model, as it precludes it from capturing correlations in the neural activity induced by the encoding distribution. In this case, lowest limiting values of the distortion are not reached for *any* values of the target rate, and the model is severely limited at high rates (because the narrow tuning curves obtained in the encoder model in effect induce strong anti-correlations in the neural activity, see insets of Fig. 3D). We summarize our study of the role of constraints on the generative model in lines 458-461 of Results, in lines 714-727 of Discussion, and in Fig. S3.

Our conclusions are obviously confined to the comparison among various families of generative models, including the log-normal form suggested by the reviewer. A more systematic study would require a detailed, independent investigation of this specific questions, which, it seems to us, would extend beyond the scope of the present paper.

2. One potential strength of the VAE approach is it allows for optimization of more complex aspect of the neural codes. In the current manuscript however, only location and width of the tuning curves are allowed to change. Can the shape of the tuning curve itself be optimized within a general parametric family? This will allow for a much stronger test of theory, for example, does log-normal tuning curve (Nover, Anderson, DeAngelis 2005) naturally emerge when the model is applied to motion?

The reviewer is correct in stating that the choice of the parametric form of the encoder represents an inductive bias in the model: it constrains the tuning-curve shapes that result from the optimization process. We decided to focus on an encoder made up of simple, bell-shaped tuning curves because these are widely observed in sensory neurons. (Generating narrow or complex tuning curves requires the use of strong or numerous synapses, so it is plausible that the bell shape reflects metabolic constraints.)

Having said this, as the reviewer suggests it is interesting to consider more flexible options. (And, indeed, in recent work (Blanco Malerba et al., under review) we consider encoding properties of complex tuning curves in a different context.) At the most flexible extreme, the tuning-curve shape is left completely unspecified. For example, we can set $q(r_i = 1|x) \propto f_i(x)$, with $f_i(x) = DNN(x)$, a universal function approximator (e.g., a deep neural network). In a preliminary numerical study, we found that this model did not yield a superior performance. A more detailed study will be needed to clarify the issue thoroughly, but we believe that the explanation of this results is that the constraints imposed on the decoder (generative model) enforces simple encoding tuning curves. In other words, in the presence of a constrained decoder, it is not advantageous to use a complex encoder.
It is also possible to consider encoding models with intermediate flexibility. This is particularly relevant in the context of the question raised by the reviewer on an application of the model to motion stimuli. Here, we carried out a numerical study with an encoder that can interpolate between Gaussian and log-normal tuning curves. There are various ways to achieve this; we chose a specific scheme, in which we set $q(r_i = 1|x) \propto f_i(x)$, with $f_i(x) = A_i\, exp\left(-\frac{(g(x)-c_i)^2}{2w_i^2}\right)$ and $g(x) = \alpha x + (1-\alpha)\, log(x)$. Here, the parameter α is trained along the other parameters in the model. For the distribution of stimulus speed, we employed a power law (see, e.g., Ganguli and Simoncelli, 2014). Maybe surprisingly, we obtained tuning curves

that were neither purely Gaussian nor purely log-normal, with the value of α close to 0.5. However, we found the optimum in α to be very shallow: the model performance was comparable to a model with purely log-normal tuning curves or purely Gaussian tuning curves. This suggests that the result is not robust, and additional constraints (e.g., a preference for sparse neural activity) can strongly affect the shape of tuning curves. We leave a detailed study of this issue for future work.

The current result in Fig. 8 can also be obtained using simpler framework such as in Ganguli & Simoncelli 2016.

As the reviewer states, relations between the stimulus density and neural parameters such as tuning width or density can be obtained in the efficient coding framework of Ganguli and Simoncelli. A similar relation between the stimulus density and the perceptual accuracy was also found by Wei and Stoker (2013) in an alternative efficient coding approach. In the latter, the coding accuracy is measured as in the work of Ganguli and Simoncelli, i.e., by the mutual information expressed in an approximate form as a function of the Fisher information. The 'metabolic' constraint, in the work of Wei and Stocker, instead of being expressed as a function of neural parameters, was defined as the integral of the square root of the Fisher information over the stimulus space, which the authors interpreted as a measure of coding resources. The specific choice of the exponent in the constraint (square root), while advantageous mathematically as it yields a parametrization-invariant form, is arbitrary. Indeed the framework of Wei and Stocker was generalized to account for different values of exponents of the Fisher information, in both the term quantifying the coding accuracy and the constraint term (see Morais and Pillow, 2018; Prat-Carrabin and Woodford, 2021).

In sum, results of the kind shown in Fig. 8 of our manuscript, which relate the discriminability to the stimulus density, emerge quite generally from the optimization of a function composed of two terms–one term accounting for coding accuracy (e.g., a function of the Fisher information), and the other term representing a resource constraint (e.g., a different function of the Fisher information). A broad range of choices of the specific forms of the two terms yields a power-law relation between discriminability and stimulus density. Furthermore, the same value of the exponent can be obtained from distinct choices (Morais and Pillow, 2018; Prat-Carrabin and Woodford, 2021).

There are, however, important conceptual and technical distinctions between the kind of approach we propose in our work and the efficient coding framework. First, Ganguli and Simoncelli imposed an arbitrary but highly stringent constraint in their optimizing procedure to obtain results that captured data; namely, they constrained optimization to be over a single scalar function, while in its natural setting the problem is higher-dimensional. (Direct optimization of the higher-dimensional problem yields pathological solutions with arbitrarily narrow tuning curves.) We discuss this point in some detail in the S3 Appendix section. By contrast, in our case, once the permissible parametric forms of the encoder and decoder are defined, no additional constraints have to be imposed to identify non-trivial minima in the optimization problem.

Second, and possibly more importantly, as we mention above, in efficient coding frameworks the detailed form the result depends on the specific choice of the two components of the function to be optimized. By contrast, the VAE approach is an unsupervised approach: the optimization function is a variational approximation to the maximum likelihood principle, i.e., it requires that the generative model produces samples distributed according to the stimulus

density (or as close as possible to it). By the same token, the form of the 'cost term' (rate term) in the optimization function emerges from this prescription and is not chosen arbitrarily.

Third, and equally importantly, the typical efficient coding approaches assume ideal decoding ('Bayesian inversion'), and this requires full knowledge of the prior density over stimuli. This is an idealization, as in most natural situations the knowledge of the prior is accessible only through examples (observations). Therefore, a complete theory ought to prescribe how optimal solutions are obtained on the basis of only samples from the environment (without necessitating the full knowledge of the stimulus prior). This is what our proposed approach achieves: it does not rely on the knowledge of the prior, and finds solutions on the basis of a dataset alone through a learning procedure. We note that there exist work addressing the questions of whether and how sparse coding (Rozell et al., 2008; Barello et al., 2019) or efficient coding solutions (Benjamin et al., 2022) can be obtained from learning procedures based on a set of stimulus samples in neural networks. In our case, the simple parametrization of the encoder allowed us to draw conclusions about optimal neural parameters. In future work, it would be worthwhile to contrast these various models more systematically.

To summarize, one aim of the section pertaining to Fig. 9 is to show that our approach yields a similar scaling of the error (or discriminability) as in classic efficient coding frameworks. The VAE model differs conceptually and technically from efficient coding models as described above, but its results are also consistent with experimental data. Furthermore, we illustrate the fact that a family of models can be obtained, corresponding to different values of the target rate, which predict similar scalings despite variability in the induced tuning curves. We summarize these conclusions as well as the above discussion in the revised manuscript, in the section "Case study: neural encoding of acoustic frequencies," in the section "VAE, efficient coding and learning from examples" of the Discussion, and in the S3 Appendix. We are grateful to the reviewer for having prompted us to provide more detailed comments on the relation and differences between our approach and classic efficient coding approaches; this adds quite a bit to the conceptual clarity and thoroughness of the paper.

Other Comments:
3. There have been studies in the literature (e.g., Wang, Stocker & Lee 2016; Park & Pillow 2020; Schaffner et al., 2023) on optimal encoding for different loss function in the decoding. These results are quite relevant for the current paper and should be discussed. For example, the γ in Fig. 7 is closer to 1 presumably because the objective for VAE is closer to a loss $L2$ rather than the standard infomax.

We thank the reviewer for this reminder of existing papers and for prompting us to discuss in greater detail similarities and differences between our approach and previously proposed ones (in particular, approaches proposed in the papers suggested by the reviewer). As we discuss above, in our model both terms of the optimizing function are determined by the requirement of optimizing a generative model conditional on a recognition model, while in previous frameworks the objective function and the cost term were chosen separately. Moreover, in our approach the model is learned on the basis of data samples (observations), and thus does not require the full knowledge of the prior distribution, as opposed to previous approaches in which the use of an optimal decoder relies on knowing the full form of the prior over stimuli. Specifically, the distortion term (in the Gaussian decoder case) can be viewed as a generalized L2 loss that also accounts for the variance of the decoder distribution; alternatively, it can be viewed as a variational approximation to the infomax objective function (Eq. (23)).

On the specific question of the exponent γ, raised by the reviewer, we mention that in efficient coding models the value of this exponent is set by the specific choices of the forms of the objective and cost terms in the optimizing function. In our model, different factors affect the optimal allocation of coding resources, and thus the values of this exponent. In particular, its value changes as a function of the rate, and depends on the interplay between the stimulus distribution and the decoder distribution (e.g., compare Figs. 7 and 8 with Figs. S7 and S8). Thus, the flexibility in previous frameworks to choose different forms of the optimizing function is translated here in using different inductive biases (parametrization choices) in our model.

In the revised manuscript, we devote a new section of the Discussion, titled "VAE, efficient coding and learning from examples", to a discussion of the relation between our model and the previously proposed efficient coding frameworks; in particular, the literature mentioned by the reviewer and the paper by Prat-Carrabin and Woodford (2021). We also comment on related questions in the Abstract and in the Introduction (lines 51-54 and 67-72).

4. After training the VAE, since the encoding model $p(r|x)$ is directly accessible, it seems straightforward to compute Fisher information analytically, which will make comparisons to previous results and computing discrimination threshold (Fig. 8C) more direct, instead of relying on the proxy through MSE.

We thank the reviewer for this comment, which induced us to illustrate an interesting feature of our framework. Our framework represents a case in which the Fisher information does not fully capture coding performance. This is due to the fact that our decoder, constrained by the generative model, is non-ideal and, in general, is biased, especially at low values of the rate, where neurons are highly noisy. In addition, we are considering finite-sized populations. As a consequence, the MSE does not saturate the Cramer-Rao bound. In the revised manuscript, we have added a passage in the Materials and methods section in which we calculate the Fisher information of the encoder, Eq. (19), and a Supplementary Figure to illustrate the comparison between the Cramer-Rao bound the actual decoding error (Fig. S6).

5. [Line 170] The connection between VAE and efficient coding through the rate-distortion interpretation is quite important for the paper. Some of the results discussed here should be moved to the results or the introduction section of the paper.

We thank the reviewer for this suggestion. In the revised manuscript, we now emphasize the analogy in the Introduction (lines 44-54) and in the first section of Results (lines 347-355).

6. [Line 415] Should the reference be Fig. 4C instead of 4B?

We have corrected this typo.

7. [Line 574 - 604] I am comfortable with interpretation the rate quantity R as an abstract form of constraint, but not sure if it makes sense as "deviation from spontaneous activity". As in Ref. 18 of the manuscript, spontaneous activity itself can be changed through learning and development, so it does not feel as fundamental as things such as metabolic constraint.

We agree with the reviewer that the interpretation of the rate as a deviation from spontaneous activity is speculative. Maybe the reviewer also worried about the interpretation of the marginal distribution over the neural activity as characterizing 'spontaneous activity'—and we agree

that assuming this is an unfounded leap. In the revised manuscript, we have removed any reference to "spontaneous activity" and only interpret the rate as an abstract form of constraint, as also mentioned by the reviewer (see lines 271-275 and 797-839).

(It is indeed still natural to think of the rate as an expression of a metabolic cost: the rate achieves small values when the stimulus has little impact on the distribution of neural activity (i.e., when all stimuli evoke a distribution close to the marginal distribution). Any metabolic constraint on resources employed for encoding takes this form, as it quantifies the impact of the stimulus on the neural activity. This line of thought can be made more systematic by invoking results in non-equilibrium statistical mechanics. A recent study has shown that the magnitude of the response of a system to an external perturbation is bounded above by the Kullback-Leibler divergence between the probability distributions describing the perturbed and unperturbed system (Dechant and Sasa, 2020). Thus, if we view the marginal distribution as describing the unperturbed state of the neural population, the rate term provides an upper bound on the magnitude of the response, i.e., the change in firing rate, of the neural population upon a stimulus presentation. In turn, this quantity is proportional to the spiking metabolic cost. In the revised manuscript, we have clarified this point and we now provide a more detailed discussion of the interpretation of the rate as a metabolic cost (see the revisions in the Discussion section "Interpretation of the resource constraint").

Reviewer 2:

Previous theoretical studies of efficient coding have been based on optimizing tuning functions for a given stimulus distribution under the assumption that decoding is optimal, which allows the objective function to be specified in terms of Fisher Information. The present study takes a different approach by optimizing tuning functions of the encoder simultaneously with the network parameters of a decoder, which has the architecture of a variational auto-encoder.

This is a polished manuscript describing a substantial and thorough evaluation of the proposed model. However, there is room to make clearer what contribution the findings make to the field. One key conclusion seems to be that, when the rate (R) is sufficiently high – meaning that the decoder can make use of stimulus-evoked variation in neural activity – the optimal tuning comes to resemble that obtained in previous studies (e.g. Ganguli & Simoncelli) that assumed an optimal decoder. For lower R, the solutions become degenerate and the ability for the decoder to reconstruct the stimulus declines. The former state seems to better correspond to neurophysiology, so what conclusions about the brain can be drawn from the latter?

We thank the reviewer for this question, that led us to discuss more thoroughly the differences between our approach and the previous ones. As discussed in the answer to Reviewer 1, one main feature of the VAE approach is that the optimal neural configuration is learned directly from a data sample (observations), in the absence of the full knowledge of the stimulus distribution (required, by contrast, in efficient coding approaches). The price paid for this is the dependence of the solution on different constraints; namely, the choice of the classes of permissible encoder and decoder models, and the value of the rate. Our framework allows for greater flexibility: we obtain a family of solutions on the use of neural resources, which might be helpful in capturing different brain systems. For example, the value of the constraint might be different for different sensory areas, or brain states. In previous frameworks, a similar flexibility requires changing altogether the forms of the objective function and the cost term. (On this and related issue, see also the response below and our response to Reviewer 1.)

In connection with the specifically question of the reviewer on the regime with low values of the rate, we suggest that intermediate and low rates do have a biological relevance. In the corresponding regime, we obtain broad and overlapping tuning curves—a picture consistent with the physiology of neurons in many sensory areas, as opposed to the high-rate regime characterized by narrow tuning curves. Furthermore, we note that in the work of Ganguli and Simoncelli, the overlap of tuning curves is imposed by hand (by constraining the optimization to take place only over a single scalar function), to avoid the pathological solution of extremely narrow tuning curves. (This pathological solution follows from, e.g., maximizing the Fisher information in the absence of further constraints; see, e.g., Zhang and Sejnowski, 1999.) In our framework, intermediate values of the rate yield broad tuning curves without the necessity to impose further constraints.

In the revised manuscript, we emphasize this important point in the Introduction (lines 567-69), and in the Discussion section “Optimal tuning width” (lines 773-796). We have also included a new Results section, titled “Generalization and role of the rate as regularizer” (lines 487-522), where we show that intermediate and low values of the rate also present the advantage of resulting in smoother approximations of the stimulus distribution and mitigating the risk of overfitting in the case of finite training set, while still achieving low coding errors. This material, together with the results presented in Fig. 3C, point to the relevance of the intermediate- and low-rate regime.

Two significant simplifications in the modelling could benefit from further comment. The impact of basing the model on only a very small number of neurons is investigated to a limited extent, but it would be helpful for the authors to make predictions for scaling up the model based on that investigation – in particular, I wonder if the degeneracy of solutions observed at low R would dissipate also as N was increased?

We thank the reviewer for this question which encouraged us to characterize the role of the population size more thoroughly. By increasing the number of neurons, the number of available activity patterns in the population increases. At low rates, the population carries little information about the stimulus, and thus we obtain degenerate solutions even with a small number of neurons. If we define the crossover population size, $N^*(\bar{R})$, as the one that saturates the lowest limiting value of the distortion for a given value of target rate, $\bar{R}$, (i.e., reaches a point on the ideal line, $D = H - \bar{R}$), then for N larger than $N^*(\bar{R})$ the degeneracy of the solutions is enhanced. Indeed, since the target rate imposes an upper bound to the mutual information of the population, adding neurons does not raise the informational content of the population activity. Thus, by increasing N beyond $N^*(\bar{R})$, the number of encoder solutions yielding the same value of the ELBO increases. As $\bar{R}$ increases, $N^*(\bar{R})$ increases; correspondingly, the degeneracy in the number of solutions that yield the same value of the ELBO is decreased for a fixed *N*. In the revised manuscript, we have updated and expanded Fig. 5, and we have added a passage in the corresponding section to illustrate this phenomenon. We also now comment on this issue in the section “Analysis of the family of optimal solutions” (lines 429-436).

The other assumption is that each unit spikes once or not at all – arguably a neurophysiological "decoder" would integrate spikes over some longer time period: do the authors think this simplification could have influenced their results?

The main motivation for assuming binary neurons was that it enabled us to enumerate all the possible neural activity patterns, and thereby compute the distortion, *D*, and the rate, *R*, without

having to introduce further approximations. In general, this assumption is valid in scenarios in which sensory coding occurs on a short time case, or when decoding neurons operate on a short time scale (e.g., have a large leak conductance); in such cases, the activity of encoding neurons over short time windows matters, during which the probability of firing more than one spike is negligible. Indeed this kind of scenario has been the object of a large number of studies in computational neuroscience; see, e.g., work on maximum-entropy models (e.g., Schneidmann et al., 2006; Tkacik et al., 2010) or generalized-linear models with binomial link-function (Runyan et al., 2016; Kira et al., 2022). We comment on the relevance and implications of this assumption in the manuscript (lines 88-90 and 94-97).

It would be interesting to explore the implications of relaxing the assumption of binary neurons. With a fixed number of neurons, allowing more than one spike per neuron increases the number of possible neural activity patterns. Thus, in the regime in which solutions are degenerate, relaxing the assumption of binary neurons would increase the number of solutions that yield the same coding performance, i.e., increase degeneracy. An important question would be whether this would allow for more solutions with broad tuning curves, which we expect to occur. We prefer to leave this study for future work, however, as relaxing the assumption of binary neurons will involve some amount of non-trivial technical work to account for an exponentially larger number of possible activity patterns in the neural population.

In principle, even with binary neurons it is possible to obtain a solution with two or more identical tuning curves. This would effectively correspond to a single ‘super-neuron’ with a binomial distribution of spikes. This kind of solutions are allowed in our setup. In our numerical simulations, however, we did not observe this kind of solution, even though tuning curves overlap considerably in the regime of intermediate rate values.

In the revised manuscript, we comment on the relevance and implications of the assumption of binary neurons. We mention possible biological motivations (in particular, the need for rapid coding in various natural situations); we also emphasize that, even with the assumption of binary neurons, the generative model is highly flexible (lines 120-129 and 681-687).

My remaining comments suggest ways in which the clarity of presentation could be improved:

Fig 1: the legend refers to rate and distortion terms, but there are no corresponding labels in the figure. It is not clear what is referred to as “a Boltzmann machine” in the legend (the decoder?) – either clarify or remove this reference.

We thank the reviewer for pointing out these issues. We have now entirely redone Fig. 1 to make it both clearer and more thorough.

“The generative model maps neural activity...to parameters of a Gaussian distribution over stimuli” – I understand this is a typical use of the term in ML but in the cognitive sciences “generative model” usually refers to an internal model of how latent states map to stimuli. For the understanding of readers who expect the latter meaning, please address this potential confusion directly at an early point in the manuscript.

We thank for the reviewer for this remark. In response to this comment, and taking into account a similar observation made by Reviewer 4, we have rewritten the passages in which we introduce the generative model approach, both in the Introduction (lines 21-33) and in the Materials and methods section (lines 78-168) to improve the clarity of the exposition.

Do color patches in Figs 4B,D,E reflect variability across different initializations of parameters, e.g. Ses? Do optimal tuning functions/parameters illustrated in Figs 4C, 8B etc reflect a single initialization or are they averaged in some way? It is hard to assess to what extent the variation with R in these plots reflects real changes in the optimum versus random differences in initialization of simulated parameters. Similarly in 8C it is difficult to interpret the fits without an indication of the uncertainty due to simulation noise.

In the Materials and methods section and in the figure captions under scrutiny, we now describe more clearly the quantities that are shown. In the revised manuscript, we now also illustrate the variability of the RMSE across different parameters initializations, in Fig. 9C (formerly Fig. 8C).

For the numerical simulations, the initialization of encoder parameters \theta is described, but not the decoder parameters \phi. Please explain.

The parameters of the deep neural network decoder were initialized using the standard method for a linear layer in PyTorch, which corresponds to Kaiming initialization; we have now clarified this point in the revised manuscript (lines 313-319).

Reviewer 3:

The authors propose a novel approach to efficient coding, expanding the usual scheme to include a separate encoding and decoding structure. Here they use a simple tuning-curve-based encoder and a flexible decoder, in contrast with the more common approach to use a complex encoder and a simple decoder (or a simple encoder and simple decoder). The paper describes degeneracies in the results, and explores how the family of solutions are parameterized by a rate-distortion tradeoff. They also compare performance to psychophysical sensitivity in an auditory discrimination task.

Overall I find this to be a nice paper, providing a new perspective on a classic problem, and deriving interesting behaviors from appropriate principles. It sets up an effective combination of simplicity of analysis and modest complexity from a shallow two-layer neural network. The resultant optimized tuning curves have some surprising behaviors. I especially like their principled derivation of the ß-VAE.

Concerns:

For me the most important missing ingredient is any addressing of generalization. Generalization is THE critical property that motivates generative models, and models with different inductive biases (such as they uncover as a function of the rate-distortion tradeoff) should generalize differently.

Concretely, in the scalar case, the authors seem only to evaluate performance on sampled training data. It seems that the values of their otherwise degenerate solutions would be different if they evaluated performance on resampled testing data. The smoother solution of Figure 3, top, may well generalize better to new data than the wiggly solution of Figure 3, bottom. The authors claim that these are equally accurate (L312), but that seems implausible to me based on their curves. I suspect that generalization of these sorts is a pretty minor effect when we're talking about scalar stimuli, so it may be that they need to move to a more complex framework

to demonstrate bigger impacts of their tradeoffs for generalization. Nonetheless, since this is such a fundamental issue it should be discussed.

We are very grateful to the reviewer for the suggestion, and the issue of generalization is certainly an important and interesting one, and it was definitely an omission not to have it discussed in the previous version of the manuscript. The reviewer's comments prompted us to conduct further investigations, leading to new results that greatly improved the manuscript.

We addressed the problem as follows. In the previous version of the manuscript, all results were obtained in the asymptotic limit of large training set, such that when evaluating the model on a separate test data set, the performance (whether it was quantified through the rate, the distortion, the ELBO, or the likelihood of the generated data) was indistinguishable from the one obtained on the training set. In the revised manuscript, we address the question of generalization by reducing the size of the training set, and exploring how the performance on the test data set differs from the asymptotic limit described above, i.e., we study the "generalization gap" in various numerical examples.

We obtained interesting results, and these illustrate the fact that generalization is an important question even in the simple scenario of a scalar stimulus we consider in the paper. In order to characterize the generalization gap, we chose to focus on the distortion measure, since the rate term was less sensitive to variations in the training set size and always close to its asymptotic value. First, we found the that the distortion on a finite training set was smaller than its asymptotic value. This effect was stronger for higher rates and smaller training sets, suggesting that, at high rates, the model overfit the training samples. Indeed, we found a large generalization gap at high rates. This finding was confirmed by examining the Kullback-Leibler divergence between the stimulus and the generative distribution (or, equivalently, examining the likelihood of generated samples): as suggested by the U-shape in Fig. 4B, this quantity increases at higher rates. Intuitively, as noted by the reviewer, this is because the generative model overfits the training data, yielding a jagged approximation of the stimulus distribution.

Our results taken together suggest that the rate can be viewed as fulfilling the role of a regularizer: a more stringer constraint on the rate prevents overfitting in the case of finite training sets. Correspondingly, at lower rates broader tuning curves and smoother generative distributions are learned; the resulting smoothness helps mitigate the impact of limited volume of training data.

In the revised manuscript, we now devote an entire, new section, titled "Generalization and role of the rate as regularizer," together with the new Fig. 6, to discuss the new results. Moreover, we comment on the role of the rate as a regularizer in the Discussion (lines 732-736).

I think that their explanation of their system could be improved. In particular, I had to read through the paper multiple times before I understood the logic of their approach, and even though I am quite familiar with generative models in neuroscience.

We apologize for the potential confusions in our explanation. Following the reviewer's remark, and taking into account similar remarks by Reviewer 4, we have rewritten the description of the model, in the Materials and methods sections, to improve the clarity of the exposition. In particular, we now describe both the approach that takes the encoder as a starting point (more

common in neuroscience, e.g., in discussions of efficient coding) and the approach that takes the decoder as a starting point (more common in machine learning and in a portion of the computational neuroscience literature influenced by machine learning). We have also rewritten the section titled "Training objective" to illustrated more explicitly the connections between our approach and the original presentation of the VAE by Kingma and Welling.

Relatedly, I found that their Figure 1 was not as helpful as it could be. Perhaps one option would be to provide more text within the figure to articulate the goals of the optimization, and the nature of the incompatible approximations.

We thank the reviewer for this suggestion. We have now entirely redone Fig. 1, together with an expanded caption, in order to make it both clearer and more thorough.

One thing that was unfamiliar to me was that their encoder is so simple, using only tuning curves. It seems quite different from such models as the Helmholtz Machine (HM), where the discriminator has comparable sophistication as the generative model, so it makes sense for them to train each other. Additionally, their approach only aims to match the overall stimulus distributions, rather than the latent variables themselves. It seems that these issues could be explained more thoroughly and pedagogically. Relating their approach to more approaches by others (such as the HM or other generative models) would be useful.

We thank the reviewer for suggesting a comparison of our work with other frameworks based on generative modeling. We note that there are several analogies between our model and the classic Helmoltz machine (HM), as well as more recent extensions of it (see, e.g., Vertes and Sahani, 2018). First, the generative model we employ, in its Gaussian form, is a generalized version of the classic HM with a single layer, and belongs to the exponential family analyzed by Vertes and Sahani (2018). One main difference consists in the nature of the stimulus): a one-dimensional, continuous variables in our case, and high-dimensional, binary variables in the classic HM. Furthermore, in our case the generative distribution is also an exponential family distribution, whose natural parameters are functions of the latent variables (though we write the parametrization in terms of mean and variance, not natural parameters of a Gaussian), with the difference that the relation is linear in the classical HM (while we use a deep network); correspondingly, we use both x and x^2 as summary statistics, instead of just x in the classic HM.

Second, the recognition model in the classical HM is closely analogous to our encoder. In both cases, the latent state is a binary vector, and the probability of one unit taking the value 1 (vs. 0) is obtained by composing a sigmoid function with a parametric function of the stimulus. In both cases, the latent units are conditionally independent given the stimulus. The difference between the two models lies in the nature of the parametric function of the stimulus: a linear projection in the case of the HM, and a quadratic function in our case (which is in fact a particular case of the more general formulation of Vertes and Sahani, 2018).

Thus, our model exhibits several analogies with the HM. Regarding the relative complexities of the two models, it is true that, in our case, the generative distribution is more complex (flexible) than the encoder, as it is described by an exponential family with two summary statistics (x and x^2, vs. r only) and with natural parameters obtained as 'arbitrarily complex' non-linear functions (the outputs of deep networks) of the latent variables (as compared to quadratic functions of the stimulus in the encoder case). Still, we found that these constraints are sufficiently stringent to affect the expressivity of the generative model in a non-trivial way.

Finally, regarding the latent variables, in the training algorithm of the HM, during the “sleep phase,” parameters are updated such as to minimize the Kullback-Leibler divergence between the distribution over latent variables (defined by the recognition model) and the distribution defined by the generative model. Similarly, in the VAE, the rate term is minimized when the distribution of latent variables according to the recognition matches the marginal distribution of latent variables according to the generative model. The difference is that, while in the wake-sleep algorithm parameters of the recognition model and of the generative model are updated in an asynchronous way during two different phases, in the VAE model all parameters are updated at the same time using backpropagation.

In the revised manuscript, we have included new passages in the Materials and methods section (lines 125-129 and 159-162) and in the Discussion (lines 737-754), where we discuss the connections of our approach to other types of generative models that have been introduced in the neuroscience literature, and to the HM in particular.

I find the application to auditory data unconvincing. There are not enough controls or comparisons to alternative hypotheses to learn much about whether humans are consistent or inconsistent with the authors’ theory. For example, their results are robust to various max allowed rates, which makes me wonder if these results not also compatible with conventional efficient coding?

Additionally, I’m not sure that the authors have correctly compared their theory to data. In Figure 8C the authors compare limens to performance estimated from their model. However, I am concerned that they may have made a unit error that would substantially affect their match. In particular, just noticeable differences between stimuli are measured in units of the stimulus — here, frequency. Yet they compare this directly to Mean Squared Error, which has units of stimulus^2. Shouldn’t they use RMSE (root mean squared error)? If so, this would change the slope in Figure 8C by a factor of 1/2, which would destroy the agreement between their model and data.

We thank the reviewer for this comment, which has helped us address discrepancies in the results, and improve the quality of the exposition.

First, we have identified an error in the simulations, related to an incorrect normalization of the power-law distribution which resulted in a suboptimal fit of the stimulus distribution. We have now rectified this issue, and the correction improved the quality of the fit of the stimulus distribution (see the insets of panel A in Fig 9).

Second, the reviewer is of course correct is stating that we should be using the root mean-squared error (RMSE) in the comparison with data (instead of the mean-squared error, MSE). We had indeed plotted the RMSE, but it was mislabeled as MSE.

Following the correction mentioned above, the distribution of frequency-difference limens obtained in our model (using the RMSE as a proxy) yielded a poor fit of the experimental data. Following a suggestion by Reviewer 1, in the revised manuscript we now also explore additional choices of the family of densities characterizing the decoder; in particular, log-normal densities. We found that this choice yielded a stronger dependence of the RMSE on the stimulus probability (signaled by a larger exponent in the power-law behaviour of the error, $\varepsilon(f) = A/\,p(f)^{\gamma}$), in the case of heavy-tailed stimulus distributions, such as an artificial log-normal distribution (see Fig. S2) or the distribution of natural frequencies. The choice of log-

normal densities in the decoder also achieves lower values of the loss function, as compared to the choice of Gaussian densities. Thus, a decoder based on log-normal densities yields a more accurate generative model of stimuli in the case of heavy-tailed distributions. This motivated us to apply this version of the VAE, with a decoder making use of log-normal densities, to fit perceptual data on frequency-difference limens, which yielded a good agreement with experimental results. In the revised manuscript, we have rewritten most of the section titled "Case study: neural encoding of acoustic frequencies" to present the new results and reflect the above discussion.

The question of the reviewer on whether similar results can be obtained in a classic efficient coding framework, and more generally on the connection between our approach and efficient coding approaches, is definitely worth discussing. We reproduce here comments we provided in response to a very similar point raised by Reviewer 1.

Similar results relating stimulus density and perceptual acuity can be derived in an efficient coding framework such as the one proposed by Ganguli and Simoncelli. Wei and Stocker (2013) also proposed a relation between the stimulus density and the perceptual performance, in an alternative efficient coding approach. In the latter, the coding accuracy is measured as in the work of Ganguli and Simoncelli, i.e., by the mutual information expressed in an approximate form as a function of the Fisher information. The 'metabolic' constraint, in the work of Wei and Stocker, instead of being expressed as a function of neural parameters, was defined as the integral of the square root of the Fisher information over the stimulus space, which the authors interpreted as a measure of coding resources. The specific choice of the exponent in the constraint (square root), while advantageous mathematically as it yields a parametrization-invariant form, is arbitrary. Indeed the framework of Wei and Stocker was generalized to account for different values of exponents of the Fisher information, in both the term quantifying the coding accuracy and the constraint term (see Morais and Pillow, 2018; Prat-Carrabin and Woodford, 2021).

In sum, results of the kind shown in Fig. 8 of our manuscript, which relate the discriminability to the stimulus density, emerge quite generally from the optimization of a function composed of two terms–one term accounting for coding accuracy (e.g., a function of the Fisher information), and the other term representing a resource constraint (e.g., a different function of the Fisher information). A broad range of choices of the specific forms of the two terms yields a power-law relation between discriminability and stimulus density. Furthermore, the same value of the exponent can be obtained from distinct choices (Morais and Pillow, 2018; Prat-Carrabin and Woodford, 2021).

There are, however, important conceptual and technical distinctions between the kind of approach we propose in our work and the efficient coding framework. First, Ganguli and Simoncelli imposed an arbitrary but highly stringent constraint in their optimizing procedure to obtain results that captured data; namely, they constrained optimization to be over a single scalar function, while in its natural setting the problem is higher-dimensional. (Direct optimization of the higher-dimensional problem yields pathological solutions with arbitrarily narrow tuning curves.) We discuss this point in some detail in the section S3 Appendix. By contrast, in our case, once the permissible parametric forms of the encoder and decoder are defined, no additional constraints have to be imposed to identify non-trivial minima in the optimization problem.

Second, and possibly more importantly, as we mention above, in efficient coding frameworks the detailed form the result depends on the specific choice of the two components of the function to be optimized. By contrast, the VAE approach is an unsupervised approach: the optimization function is a variational approximation to the maximum likelihood principle, i.e., it requires that the generative model produces samples distributed according to the stimulus density (or as close as possible to it). By the same token, the form of the 'cost term' (rate term) in the optimization function emerges from this prescription and is not chosen arbitrarily.

Third, and equally importantly, the typical efficient coding approaches assume ideal decoding ('Bayesian inversion'), and this requires full knowledge of the prior density over stimuli. This is an idealization, as in most natural situations the knowledge of the prior is accessible only through examples (observations). Therefore, a complete theory ought to prescribe how optimal solutions are obtained on the basis of only samples from the environment (without necessitating the full knowledge of the stimulus prior). This is what our proposed approach achieves: it does not rely on the knowledge of the prior, and finds solutions on the basis of a dataset alone through a learning procedure. We note that there exist work addressing the questions of whether and how sparse coding solutions (Rozell et al., 2008; Barello et al., 2019) or efficient coding solutions (Benjamin et al., 2022) can be obtained from learning procedures based on a set of stimulus samples in neural networks. In our case, the simple parametrization of the encoder allowed us to draw conclusions about optimal neural parameters. In future work, it would be worthwhile to contrast these various models more systematically.

To summarize, one aim of the section pertaining to Fig 9 is to show that our approach yields a similar scaling of the error (or discriminability) as in classic efficient coding frameworks. The VAE model differs conceptually and technically from efficient coding models as described above, but its results are also consistent with experimental data. Furthermore, we illustrate the fact that a family of models can be obtained, corresponding to different values of the target rate, which predict similar scalings despite variability in the induced tuning curves. We summarize these conclusions as well as the above discussion in the revised manuscript, in the section "Case study: neural encoding of acoustic frequencies," in the section "VAE, efficient coding and learning from examples" of the Discussion, and in the S3 Appendix. We are grateful to both reviewers for having prompted us to provide more detailed comments on the relation and differences between our approach and classic efficient coding approaches; this adds quite a bit to the conceptual clarity and thoroughness of the paper.

Minor comments:

Figure 1: I find it confusing to use \hat{x} in a posterior. The posterior is really over x, not over estimates \hat{x} (unless \hat{x} defines a new possibly wrong latent variable, which I don't think they do — and even in that case I wouldn't use \hat{x} because it has connotations of an estimate). An estimate \hat{x} would be a function of the posterior over x.

The reviewer is absolutely correct. We have fixed the notation in the revised manuscript.

Similarly, their figure uses D(x,\hat{x}) in the figure, but not in the text. How does the distortion depend on the uncertainty in q, as opposed to a point estimate xh? Why are they computing errors from SAMPLES from q(x|r), instead of errors of an estimate based on q(x|r), like the ML or MAP? Those errors will have terms arising from the sampling, which would be more error than needed based upon q.

We thank the reviewer for pointing out this issue in our notation; we have now entirely redone Fig. 1 and cleaned up the notation.

Regarding the computation of the errors, the latter is carried out in two ways in the paper, by making use of two different estimators respectively. The first estimator we consider is the maximum a posteriori (MAP) estimator (Eq. (16)), optimal in the case of quadratic errors. The second estimator we consider uses sampling from the posterior. This choice stems from the interpretation of the generative model in the VAE as the machinery available to the brain to simulate or predict stimuli; in other words, in the absence of an auxiliary machinery for constructing an estimate of a quantity based on the realization of the latent variables, the brain has to use the generative model, and thereby simulate stimuli on the basis of which a decision (estimate) is made. Naturally, it is possible to generate multiple samples, and thereby interpolate between a MAP estimate and a single sample; we decided to illustrate the two extreme cases.

We illustrate the errors obtained with these two choices in Fig. 4E. We now comment, also, on the rationale for a sampling approach to estimation, in the Materials and methods section (lines 223-226 and 468-471).

How can their wiggly generative distribution in Figure 3, bottom, have 17 peaks when there are only 10 tuning functions that are being combined?

We apologize for the confusion that our explanation might have generated. The generative distribution is a mixture distribution obtained as $p(x) = \sum_r p(x|r)\,p(r)$, and, in the Gaussian case, corresponds to a mixture of Gaussians. We model the neural activity pattern, r, as vector of binary variables; if there are N neurons, there are 2^N possible realizations of neural activity patterns. Thus, in general the generative distribution is a mixture of 2^N Gaussians. (The prior distribution r controls the probability of occurrence of different activity patterns. In the high-rate regime, the entropy of this prior is suppressed, and so is the effective number of Gaussians used in the mixture. This mechanism, together with the appearance of narrow tuning curves in the encoder, give rise to the jagged profile the reviewer is pointing to.)

Why does the distortion in Figure 4B decrease and then increase? I'd expect that with higher rates the distortion should monotonically decrease?

What is plotted in Fig. 4B is not the distortion, but the Kullback-Leibler divergence between the stimulus and the generative distribution. The U-shape is related to the issue of generalization: at high rates, the generative distribution becomes jagged and results in a poor approximation of the stimulus distribution.

L524: "In previous studies, internal models were defined by conditioning the probability of a stimulus, x, on the realization of a latent variable, z, through their joint distribution, q(z, x) = q(x|z)q(z). How the latent variable was related to a specific neural representation was not prescribed."

First, in these sampling models, z must be linear in r, which is actually a very strong constraint. Second, work from Ralf Haefner's group has done more along these lines, using an explicit model for r akin to the classic sparse coding approach of Olshausen and Field. Additionally, multiple works by Jeff Beck and colleagues has used generative models to derive specific neural representations.

It would be nicer for storytelling if their paper ended on note that discusses something like future prospects of their model, instead of trailing off with a critique of other suboptimal models.

We thank the reviewer for this comment, that lead us to rewrite parts of the Discussion. In particular, we have rewritten a portion of the section “Internal model and perception as inference” of the Discussion to clarify the differences in interpretation between our approach to generative modeling and previous ones. We have also expanded the section “What causes the degeneracy of optimal solutions” to mention possible future directions studies, for example making use of more complex generative models and biologically plausible encoders. Finally, we now conclude the Discussion with comments on the nature of costs and constraints, another topic of further study.

Reviewer 4:

This paper presents a new theoretical framework that synthesizes the efficient coding hypothesis, which argues that the nervous system seeks to maximize information about the environment, and generative modeling approaches, which seek to frame neural coding as inferring the hidden causes underlying sensory inputs under a generative model of the world. This novel approach takes the form of a variational auto-encoder, a generative modeling approach developed originally in the machine learning literature, which contains both an encoder (for approximate inference under an intractable generative model) and a decoder (the generative model itself). The work is novel and interesting, and likely to be of broad interest to the theoretical and computational neuroscience community. I enjoyed reading it, and think it is a highly appropriate for the readership of PLoS CB. Most of my comments focus on technical aspects of the work and issues of clarity and completeness, which I will outline in more detail below. Overall it is a very nice paper, and I congratulate the authors on this impressive contribution to the literature.

Detailed Comments:

--- line 6: " This hypothesis has been successful in predicting neural responses to natural stimuli in various sensory areas [2–4]." I would also cite Laughlin's seminal 1981 paper here (which as far as I'm aware is the first paper to propose an empirical test of the efficient coding hypothesis):

Laughlin, S. B. "A simple coding procedure enhances a neuron's information capacity" Z. Naturforsch, 1981, 36, 910-912.

We now cite Laughlin’s paper, as well as the recent, closely related paper by Schaffner et al. (2023).

--- line 28: "As opposed to the efficient coding approach, which prescribes a stochastic mapping from stimulus to neural activity, the generative model approach prescribes a stochastic mapping from neural activity to stimulus. This mapping implies a posterior distribution on neural activity, which can be read off from neural data." This is a complex idea that I think may be unfamiliar / confusing to some readers -- I might suggest unpacking this a bit more. (i.e., who does the generative modeling approach prescribe a stochastic mapping from neural activity to stimulus?)

We thank the reviewer for this suggestion. In the revised manuscript, we have rewritten the passages in which we introduce and motivate the generative modeling approach, both in the Introduction (lines 21-33) and in the Materials and methods section (lines 78-129).

In some of the responses below, as well as in some of the responses to Reviewer 2, we comment further on related issues.

--- line 40: "structure of a variational autoencoder (VAE) [17]."

I believe VAEs were proposed independently in two papers that appeared right around the same time, and so it might be nice to cite both of them. In addition to ref 17 (Kingma & Welling 2014), you might add:

Rezende, D. J., S. Mohamed, and D. Wierstra (2014). "Stochastic backpropagation and approximate inference in deep generative models". International Conference on Machine Learning. 1278–1286.

We thank the reviewer for this reminder. We now cite both papers when we first mention the VAE.

--- line 49: "the statistical constraint can thus be viewed as the metabolic cost of stimulus-induced deviations from spontaneous neural activity."

Perhaps this will become clear later, but it's not obvious to me that maximizing the ELBO (or minimizing KL between distributions) maps onto a metabolic cost.

We agree with the reviewer that an interpretation in terms of metabolic cost is speculative. Our motivation for this wording was the observation that the rate term is minimized when the stimulus has a little effect on neural activity, i.e. when the conditional probability is more or less constant for all stimuli and equal to the prior over neural activity. Any metabolic constraint on the encoding resources of the system would exhibit a similar structure. It is in this sense that we propose to interpret the rate as an abstract form of metabolic constraint, penalizing large stimulus-evoked activity changes. This line of thought can be made more systematic by invoking results in non-equilibrium statistical mechanics. A recent study has shown that the magnitude of the response of a system to an external perturbation is bounded above by the Kullback-Leibler divergence between the probability distributions describing the perturbed and unperturbed system (Dechant and Sasa, 2020). Thus, if we view the marginal distribution as describing the unperturbed state of the neural population, the rate term provides an upper bound on the magnitude of the response, i.e., the change in firing rate, of the neural population upon a stimulus presentation. In turn, this quantity is proportional to the spiking metabolic cost. In the revised manuscript, we have clarified this point and we now provide a more detailed discussion of the interpretation of the rate as a metabolic cost (see the revisions in the Discussion section "Interpretation of the resource constraint").

--- line 57: "Related approaches have been explored in the literature, and predictions about the optimal allocation of coding resources, i.e., the tuning curves, as a function of the stimulus distribution have been derived [6,20]".

There is a lot of other work that would be appropriate to cite here, eg papers from Wei & Stocker, Julijana Gjorgjieva's work with Meister on optimal coding in retina, Wang, Stocker & Lee 2012, Berens & Ecker et al PNAS 2011, to name just a few.

We thank the reviewer for pointing us to this literature. We now cite the papers referred to by the reviewer in the relevant passage (to the exception of the paper by Berens et al. (2011), which does not consider the optimal allocation of neural resources as a function of the stimulus distribution).

--- line 81: " We assume neurons to spike independently, such that pθ(r|x) = QNi=1 pθ(ri|x)."

Why make this assumption? (This seems to be at odds with what we know about the brain, which is that neurons exhibit non-zero noise correlations). I think it's worth giving more justification for this assumption.

There are multiple reasons for this choice. First, we ignored noise correlations for the sake of tractability. Second, we wanted to keep the encoder model as simple as possible, in order to study the optimal configurations of tuning parameters, and, correspondingly, 'signal' correlations. Modeling noise correlations would require the introduction of additional parameters ($O(N^2)$ additional parameters); we expected that this would enhance the degeneracy of the solutions (the multiplicity of configurations of parameters yielding the same performance) and, thereby, make the interpretation of our results less clear. Third, and maybe most importantly, leaving noise correlations to be completely flexible would potentially result in numerous solutions (some pathological) characterized by specific combinations of signal and noise correlations. To set up the problem in a fruitful way, one would need to constrain the class of possible correlations structures. In order to do so in a principled way, one would then have to specify how the noise correlations arise (e.g., from properties of network architecture). It seemed to us that this would require a full-fledged investigation that would lead us much beyond the scope of the present paper. It would, however, be an interesting object of future study.

In the revised manuscript, we comment on this point (lines 144-150).

Also, a minor comment: this is conditional (not marginal) independence, so it would be more accurate to say "We assume neurons spike independently given the stimulus" (or to say "conditionally independent").

Thank you. We have modified the corresponding passage (lines 141-143).

--- eq 2: With this normalization you have replaced Poisson with Bernoulli, so it seems like it would be more accurate to simply refer to this as a Bernoulli model. This approximates a Poisson distribution only in the limit of fi(x) << 1. Is that the case here? If f_i(x) / (1 + f_i(x)) ever approaches 1/2 then you are far from Poisson. (But I think it's perfectly fine to use a Bernoulli model for spike counts in a small time bin, if that's what you want here).

The reviewer is correct that our aim is to use a Bernoulli model, since a Poisson model would require a more complex prior distribution on neural activity, and it would appreciably increase the computational load due to the much larger number of possible configurations. We have now modified the corresponding passage to clarify this question: we now state that we use a

Bernoulli model, and mention in passing that it can also be viewed as a limiting case of a Poisson model.

--- line 112-114: "Thus, we model the generative distribution as a Gaussian, whose mean (stimulus estimate) and variance (uncertainty) are generic functions of neural activity patterns."

Again, I'd suggest unpacking this a bit more. The fact that the mean corresponds to the "stimulus estimate" may not be clear or obvious to all readers.

We thank the reviewer for this suggestion. In the revised manuscript, we have rewritten the passages in which we introduce and motivate the generative modeling approach, both in the Introduction (lines 21-33) and in the Materials and methods section (lines 78-129).

--- line 119: missing "of". (space "of" neural activity patterns).

Thank you. We have corrected the sentence.

--- line 123. I found the section on "Training Objective" a bit odd -- the way it's written, it sounds like you made an arbitrary choice to use KL (P,Q) as the measure of fit between the model and the data. It's completely unmotivated and seems to be an ad hoc choice of objective function. (For example, why KL (P,Q) instead of KL (Q,P)?)

I would suggest rewriting this section to follow a bit more closely the motivation and logic from the original VAE papers, namely that we'd LIKE to fit our generative model parameters by maximizing log-likelihood, but this turns out to be intractable, so instead we apply Jensen's inequality to obtain a lower bound on the log-likelihood (known as the ELBO). Then you could show how the terms that fall out from this approach map onto the quantities you want to talk about from the encoder and decoder. The way this section is currently written, it sounds like you are introducing an entirely new approach (which we only find out later has a mapping onto the well-known VAE). I think it would serve the reader better to introduce the ELBO in a more principled / well-motivated way. This would also help make clear which parts of the paper are novel, vs. building on prior work.

We thank the reviewer for this comment. Following the reviewer's remark, and taking into account similar remarks by Reviewer 3, we have rewritten the description of the model, in the Materials and methods sections, to improve the clarity of the exposition. In particular, we now describe both the approach that takes the encoder as a starting point (more common in neuroscience, e.g., in discussions of efficient coding) and the approach that takes the decoder as a starting point (more common in machine learning and in a portion of the computational neuroscience literature influenced by machine learning). We have also rewritten the section titled "Training objective" to illustrated more explicitly the connections between our approach and the original presentation of the VAE by Kingma and Welling.

One other minor quibble is that the paper reverses the standard definitions of P and Q in the VAE literature (and the variational inference literature more broadly), in a way which made the derivations harder to follow (for me at least). The original Kingma & Welling paper uses Q(x) to denote the encoder (ie the approximate posterior distribution over the latents given the data) and P(x) to denote the probability distribution of the data under the generative model. So they get KL(Q,P) as the objective. I don't know if there's some particular reason the authors here have chosen to reverse notation so that Q is the generative distribution, but I think most

readers will find it easier to connect the paper with previous literature if you keep Q and P in line with Kingma & Welling's definitions (and other VI literature where Q(x) is the approximate posterior).

We thank the reviewer for this very valid suggestion. We have now modified our notation to align it with the one used in the VAE literature.

--- line 43: "We note that the maximum value of the ELBO corresponds to minus the stimulus entropy, yielding a vanishing DKL divergence in Eq. 144 (7)."

I don't think this is quite correct -- I think the KL divergence vanishes only if the recognition model is sufficiently rich that it can accurately describe the posterior distribution under the generative model. (But if it is not expressive enough then this divergence remains non-zero). My guess is that the assumption of conditional independence in the encoder (eq. 82) will mean that this encoder is not flexible enough for the KL to vanish (for example, it cannot capture dependencies in the approximate posterior, so there is no "explaining away"), but this is just a hunch.

This can be seen from Eq. (10) in the revised manuscript, which results from the D_{KL} chain rule applied to Eq. (8): since the left-hand-side of Eq. (10) is non-negative, the ELBO is bound above by the stimulus entropy. This bound is achieved when the left-hand-side of Eq. (10), i.e., when the two joint distributions (of the recognition and generative models) match.

We have nearly entirely re-written the section Materials and methods to clarify points such as this one.

--- line 170: the section on constrained optimization is highly technical. I wonder if the authors might consider putting only a shorter high-level / intuitive summary of the approach here, and moving the technical details to an appendix (ie for experts who want them)?

We thank the reviewer for this suggestion. In the revised manuscript, we now highlighted the main steps of the analysis of the solution necessary for the logical flow, and we have moved all details to an Appendix.

--- line 444: "the error is well described by a power law".

It might be worth citing these two papers, which also describe the emergence of power laws in virtue of the choice of loss function for efficient coding (although I'm not sure if the form here is exactly the same):

- Morais, M. & Pillow, J. W. .Power-law efficient neural codes provide general link between perceptual bias and discriminability Advances in Neural Information Processing Systems 31, 2018

- A unifying theory explains seemingly contradicting biases in perceptual estimation. M Hahn, X Wei, bioRxiv, 2022.12. 12.519538.

Thank you. We now cite both these references in the context of our discussion of the power-law behavior of the mean-squared error as a function of stimulus probability.
